# Financial incentives for return of service in underserved areas: a systematic review

**DOI:** 10.1186/1472-6963-9-86

**Published:** 2009-05-29

**Authors:** Till Bärnighausen, David E Bloom

**Affiliations:** 1 Africa Centre for Health and Population Studies, University of KwaZulu-Natal, 3935 Mtubatuba, South Africa; 2 Department of Global Health and Population, Harvard School of Public Health, Harvard University, 665 Huntington Avenue, Boston, USA

## Abstract

**Background:**

In many geographic regions, both in developing and in developed countries, the number of health workers is insufficient to achieve population health goals. Financial incentives for return of service are intended to alleviate health worker shortages: A (future) health worker enters into a contract to work for a number of years in an underserved area in exchange for a financial pay-off.

**Methods:**

We carried out systematic literature searches of PubMed, the Excerpta Medica database, the Cumulative Index to Nursing and Allied Health Literature, and the National Health Services Economic Evaluation Database for studies evaluating outcomes of financial-incentive programs published up to February 2009. To identify articles for review, we combined three search themes (health workers or students, underserved areas, and financial incentives). In the initial search, we identified 10,495 unique articles, 10,302 of which were excluded based on their titles or abstracts. We conducted full-text reviews of the remaining 193 articles and of 26 additional articles identified in reference lists or by colleagues. Forty-three articles were included in the final review. We extracted from these articles information on the financial-incentive programs (name, location, period of operation, objectives, target groups, definition of underserved area, financial incentives and obligation) and information on the individual studies (authors, publication dates, types of study outcomes, study design, sample criteria and sample size, data sources, outcome measures and study findings, conclusions, and methodological limitations). We reviewed program results (descriptions of recruitment, retention, and participant satisfaction), program effects (effectiveness in influencing health workers to provide care, to remain, and to be satisfied with work and personal life in underserved areas), and program impacts (effectiveness in influencing health systems and health outcomes).

**Results:**

Of the 43 reviewed studies 34 investigated financial-incentive programs in the US. The remaining studies evaluated programs in Japan (five studies), Canada (two), New Zealand (one) and South Africa (one). The programs started between 1930 and 1998. We identified five different types of programs (service-requiring scholarships, educational loans with service requirements, service-option educational loans, loan repayment programs, and direct financial incentives). Financial incentives to serve for one year in an underserved area ranged from year-2000 United States dollars 1,358 to 28,470. All reviewed studies were observational. The random-effects estimate of the pooled proportion of all eligible program participants who had either fulfilled their obligation or were fulfilling it at the time of the study was 71% (95% confidence interval 60–80%). Seven studies compared retention in the *same *(underserved) area between program participants and non-participants. Six studies found that participants were less likely than non-participants to remain in the same area (five studies reported the difference to be statistically significant, while one study did not report a significance level); one study did not find a significant difference in retention in the same area. Thirteen studies compared provision of care or retention in *any *underserved area between participants and non-participants. Eleven studies found that participants were more likely to (continue to) practice in any underserved area (nine studies reported the difference to be statistically significant, while two studies did not provide the results of a significance test); two studies found that program participants were significantly less likely than non-participants to remain in any underserved area. Seven studies investigated the satisfaction of participants with their work and personal lives in underserved areas.

**Conclusion:**

Financial-incentive programs for return of service are one of the few health policy interventions intended to improve the distribution of human resources for health on which substantial evidence exists. However, the majority of studies are from the US, and only one study reports findings from a developing country, limiting generalizability. The existing studies show that financial-incentive programs have placed substantial numbers of health workers in underserved areas and that program participants are more likely than non-participants to work in underserved areas in the long run, even though they are less likely to remain at the site of original placement. As none of the existing studies can fully rule out that the observed differences between participants and non-participants are due to selection effects, the evidence to date does not allow the inference that the programs have caused increases in the supply of health workers to underserved areas.

## Background

In many geographic regions, both in developing and in developed countries, the number of health workers is insufficient to achieve population health goals. The 2004 Joint Learning Initiative (JLI) report *Human Resources for Health *estimated that "Sub-Saharan countries must nearly triple their current numbers of workers by adding the equivalent of one million workers through retention, recruitment, and training if they are to come close to approaching the MDGs [Millennium Development Goals] for health" [1]; the 2006 *World Health Report *concluded that " [t]he severity of the health workforce crisis in some of the world's poorest countries is illustrated by WHO estimates that 57 of them (36 of which are in Africa) have a deficit of 2.4 million doctors, nurses and midwives" [2]. In many developed countries, there are areas (commonly rural or poor communities) that are considered to be underserved with health workers (for instance, because the number of workers is insufficient to provide primary health care to all residents in an area) [3-5].^1^

Interventions intended to alleviate health worker shortages include selective recruitment and training for practice in underserved areas, improvements in working or living conditions, compulsory service, or incentives [6]. In this article, we systematically review the evidence on one specific set of interventions: financial incentives for return of service. These interventions work as follows. A health worker in training or a fully trained health worker enters into a contract to work for a number of years in an underserved area in exchange for a financial pay-off. Financial incentives can increase the numbers of health workers in underserved areas by a number of mechanisms. First, they can redirect the flow of those health workers who would have been educated without any financial incentive from well-served to underserved areas, for instance by decreasing the net emigration flow of nurses and physicians from developing to developed countries [7-9] or by increasing the net flow of physicians from urban tertiary care to rural primary care in developed countries [10,11]. This first mechanism can take hold if there are (future) health workers who normally would not work in an underserved area, but who are willing to do so in return for a financial incentive. Second, financial-incentive programs can add health workers to the pool of workers who would have been educated in the absence of such programs and place them in underserved areas. The second mechanism can take hold if, on the one hand, there are qualified candidates who would not have the means to finance a health care education without a financial incentive and, on the other hand, a country's health care education system can absorb additional students. Third, financial-incentive programs can decrease the outflow of health workers from underserved areas, if they prolong the retention times in underserved areas of those workers who participate in a financial-incentive program, but who would have worked in an underserved area even if they had not received a financial incentive. Improved retention in this group of health workers can be a direct result of the contractual obligation to remain for a certain number of years in an underserved area or can be caused by a program's additional efforts to increase retention (e.g., by increasing health workers' satisfaction with their work environment and career progression, or by increasing the satisfaction of health workers' families with their integration into the community) [12]. Fourth, the programs can decrease the outflow of participating and non-participating health workers from underserved areas by increasing the number of health workers in those areas through any of the three mechanisms described above. Such positive feedback may occur because increasing the number of health workers can diminish reasons for non-retention in rural and remote areas, such as high workload [13-15], lack of contact with colleagues [14], lack of support from medical specialists [16], or social isolation [15].

We have recently shown that a specific type of financial-incentive program, scholarships in return for a commitment to deliver antiretroviral treatment in Sub-Saharan Africa, is highly cost-beneficial under a wide range of assumptions [17]. In the following, we will first systematically review studies on financial incentives for return of service. Then, we will critically summarize the findings from existing studies and draw implications for policy and future research. One previous study has systematically reviewed the evidence on financial-incentive programs for return of service. Sempowski (2004) reviewed 10 studies of financial-incentive programs published between January 1966 and July 2002 [18]. The author concluded that "ROS [return-of-service] programs to rural and underserviced areas have achieved their primary goal of short-term recruitment but have had less success with long-term retention" [18]. Prima facie, an update of this systematic review is useful because more than six years have passed since the end of the period of publication of articles considered therein. In addition to the update of evidence, our review differs from the previous one in two aspects. First, the previous review was restricted to studies of physicians, while we consider studies of all types of health workers. Second, the previous review focused on program results (i.e., descriptions of outcomes among program participants without comparison to outcomes in non-participants) [18], while our review includes program results, program effects (i.e., analysis of program effectiveness at the individual-level through comparison of outcomes among participants and non-participants), and program impacts (i.e., analysis of program effectiveness at the population level, such as changes in physicians density or population mortality) (Table 1).

**Table 1:** Study outcomes

**Program results **(Program outcomes among participants)	**Program effects **(Program effectiveness at the individual level)	**Program impacts **(Program effectiveness at the population level)
*Recruitment*	*Provision of care*	*Health system*
What proportion of program participants fulfill their obligation to work in an underserved area? (14)	Does program participation affect a health worker's likelihood of providing care in an underserved area? (11)	Does the program affect health systems outcomes (e.g., physician density)? (6)
*Retention*	*Retention*	*Health*
What proportion of program participants continue to work in an underserved area after having fulfilled their obligation? (17)	Does program participation affect a health worker's likelihood of continuing to provide care in an underserved area after a certain period of time? (7)	Does the program affect health outcomes (e.g., mortality)? (1)
*Participant satisfaction*	*Participant satisfaction*	
What proportion of program participants are satisfied with	Does program participation affect a health worker's satisfaction with	
- work in the underserved area	- work in the underserved area	
- life in the underserved area	- life in the underserved areas? (2)	
- other aspects of the financial-incentive program? (7)		
*Family satisfaction*		
What proportion of relatives of program participants are satisfied with		
- work in the underserved area		
- life in the underserved area		
- other aspects of the financial-incentive program? (3)		

The term underserved area in the table encompasses both underserved geographical areas and underserved populations. The number of studies investigating the specific outcomes is shown in parentheses. The numbers in parentheses add up to 68. Twenty-five studies contribute 1 outcome each to this sum, while nine studies contribute 2 outcomes each, seven studies contribute 3 outcomes each, and one study contributes 4 outcomes. Two published articles report the same outcomes from the same study [41,42]; these outcomes are counted only once in the table

## Methods

### Data sources and search strategies

We carried out a systematic literature search in four electronic databases: PubMed [19] in order to cover articles on financial-incentive programs published in the medical literature; the Excerpta Medica database (EMBASE) [20] in order to cover articles in medical journals that are not included in PubMed, in particular European journals [21]; the Cumulative Index to Nursing and Allied Health Literature (CINAHL) [22] in order to cover articles published in the literature on nursing and allied health professions; and National Health Services Economic Evaluation Database (NHS EED) [23] in order to cover health economics studies. We used the Cochrane Library to search in NHS EED [23]. Because MEDLINE records were included in the search, we excluded MEDLINE records in both the EMBASE and the CINAHL search. No search option to exclude MEDLINE records was available in NHS EED. In order to detect any early financial-incentive program, we searched the literature from the earliest date at which records were available in each of the four databases given our search strategies. We searched all four databases on 31 January 2009 and included all relevant articles available in the databases up to the search date. In addition, we searched the reference lists of all publications included in the final review as well as of all articles that were excluded from the review because they were review articles, editorials, or commentaries. Finally, we asked colleagues with a research interest in human resources for health to identify articles on financial incentives for return of service.

To identify articles for review, we combined three search themes using the Boolean operator "and": health workers or students, underserved areas, and financial incentives. We combined several search terms with the Boolean operator "or" in order to operationalize the search themes. We drew the search terms from the controlled vocabularies used for subject indexing in PubMed (i.e., Medical Subject Headings (MeSH) [24]), EMBASE (i.e., EMTREE [25]), and CINAHL (i.e., CINAHL Subject Headings [26]). We used all search terms from the controlled vocabularies in their "exploded" versions. That is, in addition to the selected terms, all narrower terms that are categorized below it in the vocabulary hierarchies were included in the searches. While MeSH are available in NHS EED when searched through the Cochrane Library, we entered the search terms in all searchable, subject-specific fields (title, keyword, and abstract), because such a search strategy has been found to be superior to MeSH-based strategies in NHS EED [27]. The four search algorithms are shown in the Appendix.

### Selection criteria

Articles were considered for inclusion in the systematic review if they reported data from a quantitative study of results, effects, or impacts of at least one financial-incentive program for return of service. We considered articles published in any language. We excluded studies that evaluate programs that attempt to increase the number of health workers in underserved areas primarily through non-financial means [28-32]. For instance, studies evaluating the Physician Shortage Area Program (PSAP) of Jefferson Medical College were excluded because the program aims to increase the number of rural family physicians primarily through selective admission of candidates to medical school and through intensive exposure of the program participants to rural family practice, while offering only "a small amount of additional financial aid [...] almost entirely in the form of repayable loans", which "represents only a small portion of each student's entire tuition and expenses" (Rabinowitz et al. 2005).

Reviews, commentaries, editorials, news and policy briefs were excluded. Studies of financial incentives for return of service within the military (e.g., [33]) were excluded because experiences with return-of-service programs in the military are likely to be very different from civilian experiences, as the military can exert more control over its members than most civilian institutions over citizens. Studies of financial incentives for research positions (e.g., [34]) were excluded because health workers who conduct medical research are commonly motivated by very different factors than health workers in patient care [35], and this article's objective is to examine the evidence on financial incentives for return of patient care in underserved areas. We further excluded studies of financial incentives to enroll in a specific residency program [36], unless they were explicitly linked to work in underserved areas, and studies investigating the attractiveness of hypothetical financial-incentive programs [37].

After exclusion of 131 duplicate records, our searches identified a total of 10,495 articles, 10,302 of which were excluded based on their titles or abstracts. We conducted full-text reviews of the remaining 193 articles and of 26 additional articles identified in reference lists or by colleagues. Forty-three articles were included in the final review. While we did not apply any language restrictions in our search, all reviewed titles and abstracts were available in English (some as translations of original-language versions) and all articles included in the final review were published in English.

### Statistical analysis

We used DerSimonian and Laird meta-analysis [38] to compute both fixed- and random-effects estimates of the pooled recruitment proportion (and its 95% confidence interval (CI)). We defined the recruitment proportion as the proportion of all eligible program participants who had either fulfilled their service obligation or were fulfilling it at the time of the study. Participants were eligible if they had completed the required minimum medical training and were available to fulfill their obligation. Participants could be unavailable for a number of reasons, including disease, imprisonment, or temporary deferral of service. For the meta-analysis, both the recruitment proportion of a program and the total number of eligible program participants needed to be known. We thus could only include those studies in the meta-analysis that reported sufficient information to calculate these two measures. To determine the pooled recruitment proportion, the variances of the raw proportions were stabilized using the Freeman-Tukey double arcsine transformation [39]. After meta-analysis of the transformed variable, we retransformed the pooled mean and its 95% CI back to proportions. Heterogeneity of the recruitment proportion across studies was diagnosed with the Q test [40]. When significant heterogeneity was present, we selected the random-effects estimates.

## Results

Table 1 describes the outcomes that were investigated by the 43 studies included in the review and the number of studies investigating each outcome (in parentheses). Twenty-five studies investigated 1 outcome; nine studies investigated 2 outcomes; seven studies investigated 3 outcomes; and one study investigated 4 outcomes. Two published articles report the same outcomes from the same study [41,42]; these study outcomes are counted only once in Table 1.

Additional file 1 shows descriptions of each of the programs that were evaluated in at least one of the included studies. When information on some program characteristics was not available in the reviewed study itself, we extracted the information from other sources (shown in the column "Other sources" in the table). All monetary values in the column "Financial incentive and obligation" are shown both as they are provided in the reviewed study and – for ease of comparison – in year-2000 United States dollars (USD). We used the purchasing power parity index from the World Bank Development Indicators [43] in order to translate the values of a non-US currency into US dollars and the consumer price index from the US Department of Labor Bureau of Labor Statistics [44] to adjust for differences in the real value of one USD over time.

The programs evaluated in the studies included in this review started between 1930 and 1998. With the exception of five programs that accepted a range of health professionals (the North Carolina Rural Loan Program, the National Health Service Corps (NHSC), the West Virginia Recruitment and Retention Community Project, the West Virginia State Loan Repayment Program in the US, and the Friends of Mosvold Program in South Africa), the financial incentives of the evaluated programs were targeted only at future or current physicians (Additional file 1).

With the exception of three programs that cover, respectively, "tuition, entrance and equipment fees and living expenses" [45],“tuition, fees” and “a living stipend" [46], and "funds for university tuition, books, residence fees and food" [47], the precise monetary values of the financial incentives of all programs included in this review were available in published articles or on web pages. The financial incentives per year of service ranged from year-2000 USD 1,358 to 28,470. One study compared the average award amount across five types of programs – service-requiring scholarship programs, service-option educational loans, loan repayment programs, direct financial-incentive programs, and resident support (in the form of service-requiring scholarships, loan repayment, or direct financial incentives). The study did not find significant differences in the size of the financial incentives [48] (Additional file 1).

**Figure 1:**
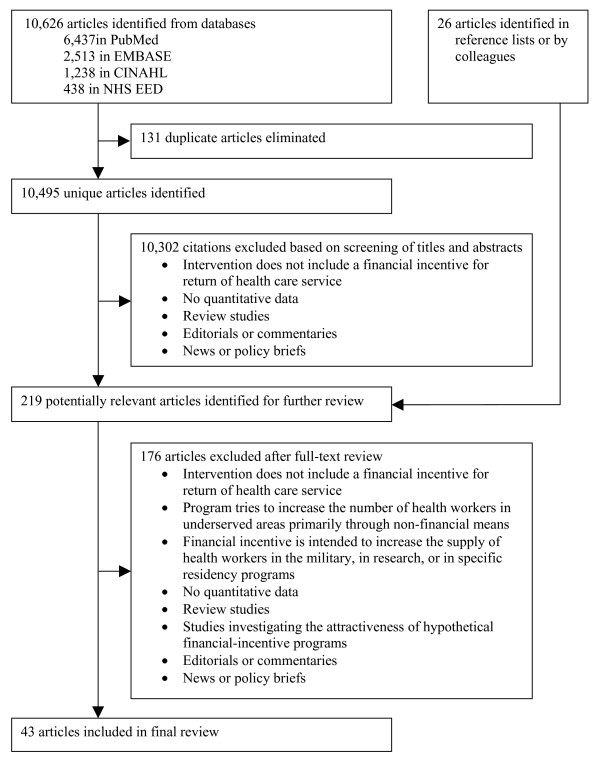
**Flowchart of the systematic review**. EMBASE = Excerpta Medica database, CINAHL = Cumulative Index to Nursing and Allied Health Literature, NHS EED = National Health Services Economic Evaluation Database.

We identified 43 studies that met all our inclusion and exclusion criteria. The previous systematic review of financial-incentive programs for return of service by Sempowski [18] identified only 10 articles, three of which were not included in our review. Two articles were not included because they evaluated a program that "tried to increase the number of health workers in underserved areas primarily through non-financial means" [49,50] (Figure 1); one study was not included because it did not report "data from a quantitative study of results, effects, or impacts of financial incentives for return of service" [51] (see "Selection criteria" above). Of the 36 articles covered in our study but not included in the review by Sempowski, 17 were published after the end of the review period of the previous study (i.e., after 2002) [11,48,52-66]. The remaining articles were not included because the previous study used different inclusion and exclusion criteria. In particular, our review considers programmatic outcomes and health worker types that were not covered in the previous study (see "Introduction" above).

Of the 43 reviewed studies, 34 investigated financial-incentive programs in the US, 24 of which evaluated the NHSC; 8 evaluated programs in specific US states or communities; 1 evaluated both the NHSC and state-based programs; and 1 evaluated the national Commonwealth Fund Medical Undergraduate Scholarship Program. Five studies investigated the Jichi Medical University (JMU) program in Japan, two assessed the Ontario Under-serviced Area Program (UAP) in Canada, and one study each evaluated the New South Wales Department of Health Rural Resident Medical Officer Program (Cadetship Program) in New Zealand and the Friends of Mosvold Scholarship Scheme (FOMSS) in South Africa.^2 ^Additional file 2 describes the study outcomes, study designs, sample criteria, sample sizes, data sources, outcome measures, effect sizes, conclusions, and methodological limitations of all studies included in the review. Sample sizes across the thirty-seven studies in which individuals were the unit of observation ranged from 24 to 493,142. Eighteen studies had sample sizes greater than 1,000, and four had sample sizes greater than 50,000.

### Types of financial-incentive programs for return of service

In our review, we identified five different types of financial-incentive programs for return of service, viz.: service-requiring scholarships (or "conditional scholarships") (e.g., [57,66]), educational loans with service requirements (e.g., [67]), service-option educational loans (e.g., [68]), loan repayment programs (e.g., [48]), and direct financial incentives (e.g., [69]) (Additional file 1). These program types differ according to the following criteria: time of commitment and time of money receipt, spending restrictions, and type of commitment. First, in the case of service-requiring scholarships, educational loans with service requirements, and service-option loans, students commit to participation in a program before or early in the course of their health care education and receive money during the education. In the case of loan repayment programs and direct financial incentives, health workers commit to participation after completion of their health care education. Direct financial incentives are commonly paid at the beginning of service in an underserved area while loan repayments are commonly made after each period of service in an underserved area (e.g., every three or six months). Second, while direct financial incentives can be used for any purpose, the money from any of the other four programs must be spent on health care education either during the education (in the case of service-requiring scholarships, educational loans with service requirements, and service-option educational loans) or after the education to repay educational debt (in the case of loan repayment programs). Finally, people who participate in service-requiring scholarships, loan repayment, or direct financial-incentive programs commit to work in an underserved area, while those receiving educational loans with service requirements commit to service and financial repayment. Individuals who receive service-option educational loans commit to either service or financial repayment. While all service option educational loans offer a choice between service and repayment of the financial incentive, some of the programs belonging to the other four types offer a buy-out option. The difference between service-option loans and service-requiring scholarships with a buy-out option is that the managers of the former will normally consider repayment and service equally desirable outcomes, whereas the managers of the latter will prefer service over buy-out. Given equal financial incentives a buy-out is thus commonly more expensive than the financial repayment of a service-option educational loan [70]. Note that many loan repayment programs do not require a buy-out option because the programs pay participants after each period they have served in an underserved area.

### Program result: recruitment

The recruitment proportion varied between 33% and 100% across programs (see Additional file 2). Fourteen studies reported for 25 different financial-incentive programs both the recruitment proportion and the total number of participants who had ever been eligible to serve their obligation (or values from which these two variables could be calculated) [45,56-58,66,68,71-73].^3 ^The random-effects pooled recruitment proportion across these 25 programs was 71% (95% CI 60–80%, heterogeneity p < 0.001).

Program participants who were available for practice, but did not fulfill their commitment to work in an underserved area, either defaulted on their obligation or bought out of it. Of the 25 programs included in the meta-analysis, only four did not offer a buy-out option [57,66,67,72]. Some programs allowed participants to repay half [74] or all [71,75] of the principal without interest in lieu of service repayment. Other programs set the buy-out price at the principal plus interest (the "prevailing rate of interest", or a fixed rate of interest varying between 2% and 10% [68]), while yet other programs charged a buyout price of the principal plus a penalty ("principal plus penalty up to 100%", or "triple the loan amount plus interest" [56]). The random-effects pooled recruitment proportion across those programs that did not offer a buy-out option (84%, 95% CI 73–92%, heterogeneity p < 0.001) was not significantly different (overall test of heterogeneity between subgroups, p = 0.652) from the pooled recruitment proportion across those programs that did allow buy-out (67%, 95% CI 55–79%, heterogeneity p < 0.001).

### Program result: retention

The proportion of program participants who remained in underserved areas after completing their obligation ranged from 12% to 90% across the eighteen articles that reported retention results [41,42,45,56,57,61-63,67,68,71,74-80]. The reported proportions, however, could not be meaningfully compared to each other, because the definition of retention, the length of time during which participants were enrolled in a study (enrolment period), and the length of time between the end of the enrolment period and the time when retention results were observed (lag time) varied widely across studies. The studies measured retention in any underserved area in a country [45,57,61,63,75,77-80], in any underserved area in a specific state [68,74], in any area in a specific state [45,56,61,62], in the underserved area of original program placement [41,42,56,67,71,77,78], or in a particular practice entered during a specific period of time [76]. Three articles reported the retention *intentions *of program participants who were fulfilling their obligations at the time rather than actual retention [41,42,79]. All of the other 15 articles described outcomes of retrospective cohort studies. One of the fifteen studies did not report an enrolment period or lag time [68]. Enrolment periods in the remaining 14 studies were four [76,77], five [71], nine [78], ten [57], fifteen [75], eighteen [63,80], nineteen [67], twenty [62], twenty-three [74], twenty-four [45], twenty-five [56], and twenty-six [61] years. There was no lag between enrolment and observation in five studies [45,56,67,71,74]; lag times in the other studies were 1 [58,63,76,80], 6 [57], 8 [78], 11 [77], and 29 [75] years; two studies assessed retention results after three different lag times (3, 7 and 9 years [61] and 9, 13, and 15 years [62]).

### Program effects: provision of care and retention

**Figure 2:**
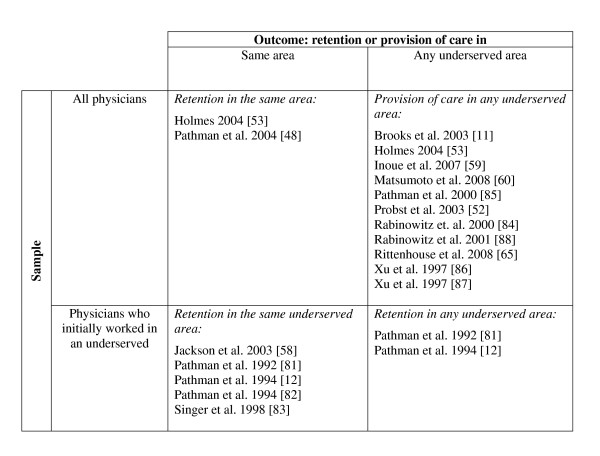
**Studies of program effect on retention and provision of care**.

In all 17 studies of program effects, program participation was defined as having received a financial incentive and serving or having served the obligation; i.e., people who received a financial incentive but could not be recruited to serve in an underserved area were excluded from the cohorts of program participants. Figure 2 shows four categories of effect studies by outcome and sample. Three categories of studies investigated retention (in the same area, in the same underserved area, or in any underserved area), and one category investigated provision of care in any underserved area. Three studies report two different program effect outcomes [53,81,82].

Five of the seven studies that compared retention in the *same *(underserved) area between program participants and non-participants found that participants were significantly less likely to remain in the area [12,48,53,81,82], while one study did not report a significance level but found a substantially higher retention in non-NHSC physicians than in NHSC physicians [83], and another study did not find a significant difference in retention between the two groups [58]. In contrast, 11 of the 13 studies that compared differences between participants and non-participants in provision of care or retention in *any *underserved area found that participants were more likely to (continue to) practice in such an area [11,52,53,59,60,65,84-88]. These differences were shown to be statistically significant in nine of the eleven studies [11,52,53,65,84-88]. Two studies did not provide the results of significance tests [59,60]. Two of the thirteen studies reported the converse finding: program participants were significantly less likely than non-participants to remain in any underserved area [81,82].

The studies of program effects reported hazard ratios [48,81], odds ratios [12,52,65,84,87,88], relative risks (or two proportions) [11,58-60,82,83,85], or beta-coefficients [53,86] as measures comparing retention or care provision among program participants and non-participants. Except for the two studies that reported hazard ratios, which took into account the duration of retention of each individual in the sample [48,81], and one study that used the proportion of underserved patients as its dependent variable [86], these studies used a binary concept of retention or care provision measured at different time intervals after an initial observation (at least 1 year [58], 1–28 years [65], 3 years and 1 month and 5 years and 1 month [12], or 5 years [83]) or after graduation from medical school (0–16 [59], 0–26 [60], 6–21 [88], 7–9 [53], 7 and 11 [85], 9–10 [84,87], 10–11 [86], 10–20 [82] or up to 29 [52] years, or an unknown time interval [11]).

### Program results and effects: participant satisfaction and family satisfaction

Seven studies investigated the satisfaction of participants with aspects of their enrolment in financial-incentive programs [12,41,48,58,67,76,77], viz. satisfaction with the overall experience in the program [48,58,67,77], satisfaction with work [12,41,48,58,76] or personal life [12,41,76] in the underserved areas, or satisfaction with aspects of program administration [58,67]. Three studies examined the satisfaction of members of participants' families with their lives in the undeserved area [41,48,76]. Four of the seven studies investigated satisfaction outcomes in the NHSC [12,41,76,77]; the three other studies examined satisfaction outcomes in US state programs [48,58,67].

While the studies on participant and family satisfaction were too few to draw any strong generalized inferences, a contrast emerged between the NHSC and the US state programs. Three studies measured overall satisfaction with financial-incentive programs in US states by asking participants whether they would enroll again in the same program [48,58,67]. They found a high counterfactual willingness to enroll again: 71% of interviewed participants in the North Carolina Rural Loan Program answered "yes" to the re-enrolment question [67]; 73% of interviewed participants in four programs in West Virginia answered either "definitely yes" or "probably yes" to the re-enrolment question [58]; and 90% of interviewed participants in US state programs indicated that they would "definitely" or "likely" enroll again [48]. In contrast, a study analyzing 183 unstructured written accounts of time in the NHSC found that only 20% of participants rated their experience as "positive", while 80% rated it either "negative", "mixed or ambivalent", or "neutral" [77].

A similar difference emerged in the comparison of NHSC and US state-based programs across specific aspects of participants' work and personal-life satisfaction (Additional file 2) For instance, in a study of state-based programs, Pathman and colleagues found that more than 80% of program participants were "satisfied with practice", more than 90% found their "work rewarding", and more than 70% felt "a sense of belonging to the community," while a comparison group of non-obligated physicians scored significantly lower on all three dimensions of satisfaction [12]. In contrast, in a study of the NHSC, Pathman and colleagues found that participants rated their satisfaction level between "dissatisfied" and "neutral" for 7 of 15 "work issues" and "personal-life" issues and participants' satisfaction level exceeded "satisfied" for only one issue (" [c]aring for needy patients"). A control group of non-participants reported significantly higher satisfaction than the participants for 9 of 15 issues (for which a comparison was made) and significantly lower for only one issue [48].

### Program impacts: health system and health

Six articles examined whether financial-incentive programs have led to changes in the number or density (i.e., number per population) of certain types of health workers [55,64,69,71,74,89]. One of the six studies described the medical student density in Arizona over time and concluded that a scholarship aiming to increase student density was not effective [74]. Two studies compared changes over time – in physician numbers (from 1966 to 1972 [71]) and in physician densities (from 1956 to 1986 [69]) – in northern Ontario to changes in the same measures in Ontario as a whole. The first study concluded that an observed increase in the absolute number of physicians in northern Ontario was likely caused by the program (because the speed of increase rose substantially after introduction of the program in northern Ontario, while there was no change in the speed of increase in Ontario overall) [71]. The second study concluded that an increase in physician density in northern Ontario was not due to the program but due to the overall increase of physicians in the province (because a measure of inequality between physician density in northern Ontario and Ontario as a whole did not improve) [69]. It is possible that an initial effect of the program in the first three years after its introduction (from 1969 to 1972) – as reported in the first study [71] – ceased to exist in the longer run (until 1986) – as reported in the second study [69]. A fourth study used data from the American Medical Association Masterfile to model the practice location choices of US physicians in sequential multinomial logit regression. The parameter estimates of NHSC participation from the regression equation were then used to predict the supply of physicians in underserved areas, assuming the NHSC had not existed. Through comparison of this counterfactual to the status quo, the study concluded that elimination of the NHSC would lead to a 10–11% decrease in the supply of physicians in underserved areas [64].

Two further studies of health system impacts of financial-incentive programs used communities as units of observation. One of the studies investigated whether underserved areas that succeeded in attracting obligated physicians were different from communities that failed to do so. It found that communities that were economically worse-off and had worse population health were less likely to receive an obligated physician than underserved communities that were economically better-off and had better population health [89]. The second study investigated whether the presence of an obligated physician in a community changed the supply of non-obligated physicians in that community and found that, when controlling for a range of demographic, economic, and health systems factors, communities staffed by NHSC clinicians experienced a larger increase in non-NHSC primary care physicians per population than communities without NHSC clinicians [55]. Only one study analyzed the effect of a financial-incentive program on a health outcome [54]. The study compared age-adjusted all-cause mortality rates in two periods, 15 years apart, in underserved communities with different levels of staffing by obligated physicians. It found no clear relationship between the level of staffing and mortality.

### Causal inferences

Causal inferences from studies reporting program results are necessarily weak, because these studies merely describe outcomes in individuals enrolled in financial-incentive programs and do not allow any comparison to individuals who did not receive financial incentives. While analyses of program effects are based on comparisons of cohorts of program participants and non-participants, causal inferences from comparisons of outcomes in different cohorts can be invalid, if there are no controls for differences between participants and non-participants.

Of the 17 studies of program effects, 11 controlled for additional variables in the comparison of retention and provision of care between people who did and did not participate in a financial-incentive program [12,48,52,53,65,81,84-88]. Eight of these studies controlled for sex of the health worker [48,52,53,65,84,86-88], five controlled for ethnicity [52,53,84,86,87], four for medical specialty [12,48,52,81], three for age [48,53,87], three for growing up in an underserved area [84,86,87], two for "strong interest" prior to medical school to practice as a doctor in an underserved area [84,87], two for childhood family income [84,87], two for characteristics of the underserved area where the health worker practices [12,52], one for marital status [48], one for the type of medical school a participant had attended (private vs. public, receiving vs. not receiving Title VII-funding [65]^4^), one for debt, experience in an underserved area during medical school, and experience in an underserved area during residency [87], one for "importance of small community living" [81], one for commitment to long-term practice in underserved areas before starting work in such an area [12], and one for expected peak income, freshman-year plans for family practice, rural preceptorship, participation in PSAP, and location of family practice clerkship [88]. Another study did not describe the particular control variables used, but reported that its effect measures remained significant "while controlling for selected characteristics of physicians" [85].

While a number of studies controlled for differences in observed characteristics between participants and non-participants, only one study of program effects attempted to control for unobserved heterogeneity in program participation. The study used a bivariate probit selection model to control for the potential bias due to selective participation [53]. In order to identify the program effect, the study used four medical school characteristics, viz. the "historical proportion of graduates specializing in primary care", the "quality of the school", a "tuition index", and a "public school indicator", assuming that these variables affected selection into financial-incentive programs but did not affect provision of care or retention in underserved areas other than through their effect on program participation. One study of program impact (by the same author) used the same medical school characteristics as identifying variables in a joint model of program participation and practice location decisions [64].

Four of the six other studies of program impacts observed changes over time in the availability of a financial-incentive program and an outcome (number or density of health workers [69,71,74] or mortality [69]), but did not control for changes over time of any other variable. Thus, in these studies it could not be ruled out that an observed relationship (or the apparent lack of a relationship) between program participation and outcome was due to a confounding variable. In addition, three of the six studies [69,71,89] may have suffered from ecological bias [90] because they observed variables at a level of aggregation that was higher than the level at which inferences were made. For instance, Anderson and Rosenberg (1990) [69] observed changes in physicians density in *counties *in order to evaluate the impact of the Ontario Underserviced Area Program in attracting physicians to underserved *communities *within those counties. Thus, the observed average change in physician density in any a county could have been caused by an infinite number of combinations of effect sizes in the different underserviced and sufficiently serviced communities in the county.

## Discussion

Of the 43 studies included in the review, 34 evaluated financial-incentive programs located in the US. The remainder examined programs in other developed countries (Japan, Canada, New Zealand), with but one exception that described a program in South Africa. The US financial-incentive programs have placed substantial numbers of health workers in underserved areas. For instance, between 1972 and 2009, the NHSC – the largest financial-incentive program in the US – placed approximately 30,000 primary care clinicians in underserved areas [46]. At the same time, the US programs have met only a small proportion of national unmet health care need. In 2008, 4,600 clinicians were serving in the NHSC, but according to NHSC estimates 27,000 additional primary care professionals were required to provide care to the 50 million people who still lacked access to primary health care in the United States [91].

While most of the evaluated programs were located in the US, the US market for health care education is unusual in comparison to many other countries in that students pay high tuition for their education. Countries where students of health care do not usually incur large debt, such as many Western European countries, may not be as successful as the US in recruiting students and health professionals into programs that provide scholarships or repayment of educational loans in return for service in underserved areas. In many developing countries, by contrast, education for a health profession can be quite costly because of tuition and school fees as well as costs of housing and living. Some of the experiences from the US may thus be more applicable to health care education markets in developing countries than to other developed countries. On the other hand, (future) health workers in the US have many options for funding their education, while funding opportunities for education may be few in some developing countries. Thus, the generalizability of US findings to other countries where students have substantial financing need for health care education may be limited because the selection into financial-incentive programs for underserved service may depend on the availability of funding alternatives. Numerous other differences, such as in the capacity to enforce and monitor obligated service (compare [92]), may limit the generalizability of the studies included in this review to other settings. One study from South Africa suggests that scholarship programs for health care education can be a successful instrument to recruit health workers for practice in rural Africa [66]. Future studies should evaluate outcomes of financial-incentive programs from other developing countries where such programs have been offered in the past or are currently offered, such as Swaziland [93], Ghana [94], and Mexico [95].

Notwithstanding the above caveats about generalizability, it is useful to summarize some of the key findings from our review. First, most of the financial-incentive programs experienced substantial losses to recruitment before the start of the service obligation. Across the 25 programs included in the meta-analysis in our review, on average about 3 in 10 participants did not fulfill their commitment to work in an underserved area. However, there was wide variation in loss to recruitment. As reported previously by Pathman and colleagues [48] and Jackson and colleagues [58], state programs in the US that committed students to service (service-requiring scholarships and educational loans with service requirements) had significantly lower recruitment proportions than state programs that committed health workers after their training (direct financial incentives and loan repayment programs). This finding is not surprising, because preferences change over time. For instance, students who found careers in primary care appealing at entry into medical school may develop a strong interest in highly specialized health care during their training, which depends on technology that is usually not available in underserved areas.

Furthermore, we find that the recruitment proportion did not differ significantly between programs that offered a buy-out option and those that did not. While this result suggests that participants who have decided not to serve their obligation will do so independent of the conditions of the program they are enrolled in, it is important to note that the proportion of participants who would have taken up work in underserved areas had they not enrolled in a specific financial-incentive program is unknown. Thus, it is impossible to infer from such comparison the relative recruitment effectiveness of different types of programs.

Second, participants in financial-incentive programs were significantly more likely to leave their sites of first practice after completion of their obligation than non-obligated health workers in comparable sites of first practice after service of similar length of time. There may be several reasons for this finding. For one, some of those health workers who – without financial incentive – find practice in any underserved area less attractive than practice in sites that are not underserved decide to enroll in financial-incentive programs and to complete their obligations. These health workers are likely to leave the underserved area after having served the obligated term. On the other hand, even those obligated health workers who find practice in underserved areas to be the most attractive career path in general may be more likely to leave their sites of initial practice than their non-obligated colleagues in the same underserved areas. Obligated health workers have less choice over the particular underserved area in which they first practice than their non-obligated peers and are thus less likely to be satisfied with their work and personal life in the underserved area. For instance, one study of the NHSC concludes that NHSC enrollees "placed in rural sites in the late 1980s experienced a site-matching process that they felt offered few acceptable sites" and "offered little opportunity to locate the bestsuited site among those offered" [12]. Financial-incentive programs aiming to achieve high retention of health workers in the sites where they fulfill their obligated service should attempt to accommodate health workers' wishes to practice in a particular underserved area to the greatest extent possible.

Third, while participants in financial-incentive programs were *less *likely than non-participants to remain in the particular underserved area of first practice, the reviewed studies suggest that participants were *more *likely to practice in some underserved area or to work with an underserved population than their peers who did not participate in a financial-incentive program. This summary finding from our systematic review is in contrast to the conclusion of the one previous review of financial incentives for return of service, which concluded that incentive programs "have achieved their primary goal of short-term recruitment but have had less success with long-term retention" [18].

Many of the analyses of retention in studies in this review compared the behavior of participants in financial-incentive programs to that of non-participants, controlling for a few observed health worker characteristics, such as sex, age, ethnicity, or marital status. However, since participants self-selected into programs, it is difficult to identify whether any difference in behavior between participants and non-participants was due to unobserved characteristics distinguishing participants from non-participants or due to program effects. It is possible that those health workers with the strongest preferences to serve underserved populations chose to participate in financial-incentive programs and that these unobserved preferences fully explain the different work and retention patterns in participants and non-participants, i.e., participants would have worked for exactly the same lengths of time in underserved areas without the incentives they received.

An ideal strategy to identify causal effects of financial-incentive programs is randomized controlled trials. However, since program participation is an individual choice, it will be impossible to randomize individuals into program participation and control arms. While it would theoretically be possible to randomize cohorts of medical students (e.g., by year of graduation or by medical school) to financial-incentive offers of different sizes, such a randomization strategy may not be politically or administratively feasible. An alternative strategy to identify causal effects involves the use of statistical models that control for selection into financial-incentive programs on unobserved individual characteristics. Two studies in this review (one of program effect [53] and one of program impact [64]) implemented selection models of program participation. The two studies used medical school characteristics (e.g., the "historical proportion" of graduates pursuing careers in primary care) to identify program effect. However, the type of medical school that students choose is likely to be related not only to the decision to enroll in financial-incentive programs, but also – independent of program participation – to the decision to work in underserved areas. For instance, students with strong preferences to work in underserved areas may be more likely than their peers with weaker preferences for such care to select medical schools with a high "historical proportion" of graduates pursuing careers in primary care, because such schools are likely to focus on medical education relevant for underserved practice. This selection may determine work location decisions, independent of any effect the medical school characteristic may have on participation in financial-incentive programs. Thus the characteristic may not be a valid variable to identify program effects. Despite the difficulty in finding variables to identify program effects in selection models, future studies using already-existing data should emphasize control of biases due to selection effects. In the absence of a valid method to control for selection into program participation on unobserved variables, studies of retention and provision of care should attempt to control for variables capturing health workers' preferences to work in underserved areas before financial-incentive programs could have influenced those preferences. Studies in this review controlled for intention to work in an underserved area prior to the decision to enroll in a financial-incentive program [84,87], for the intention to practice in an underserved area for a time period that is longer than the service obligation in a financial-incentive program prior to such practice [12], or for variables that are likely to be closely related to the preference to work in an underserved area in the absence of financial incentives, such as having grown up in an underserved area [84,87] or a predilection for living in small communities [81]. Two of these four studies found that physicians participating in the NHSC were significantly *less *likely than non-NHSC physicians to remain in the *same *underserved area where they initially took up work [12,81], while the other two found that NHSC participants are significantly *more *likely to provide care in *an *underserved area than non-NHSC physicians [84,87]. These four studies suggest more strongly than the other studies in this review that the finding that program participants are more likely than non-participants to work in underserved areas in the long run (even though they are less likely to remain at their site of original placement) is indeed causal. Nonetheless, they cannot rule out that the observed effects are due to selection on unobserved variables.

Fourth, financial-incentive programs varied substantially in the level of participant satisfaction. Participants in some programs were more satisfied than non-participants with their work and personal life in underserved areas, while the converse was true for participants in other programs. Health workers' satisfaction with work and personal life in underserved areas is important for several reasons. For one, health worker satisfaction influences retention, as has been shown in several studies [96-98], including in studies of financial-incentive programs for return of service [12,41,63]. Moreover, health worker satisfaction is associated with patient satisfaction [99] and quality of care [100,101]. Health workers are also likely to share their experiences with colleagues and may thus influence the supply of health workers to underserved areas as well as participation in financial-incentive programs. The reviewed studies offer some insight into the mechanism through which individual programs affect participant satisfaction. This evidence, based on case reports and participants' accounts, suggests that programs that achieved high participant satisfaction successfully interacted with participants during different stages of program enrolment, viz. participant selection, the matching of underserved areas to the preferences of individual participants, preparation of the participants and their families before the start of the obligated service, as well as career guidance, mentoring, monitoring of problems, and ongoing support during the service [12,48,58,66,70,77]. Detailed case studies of relatively successful and unsuccessful programs could further improve our understanding of management skills, organizational processes, and program features that increase participant satisfaction and retention in underserved areas.

Fifth, there is no clear evidence that financial-incentive programs had any significant impact on the supply of health workers to underserved areas. The results of three studies suggest that certain programs led to an increase in health worker numbers or densities, while two other studies did not find such program impacts. This discrepancy could be due to actual differences in impact between programs or over time; or they could be caused by methodological limitations of the studies. The impact of financial-incentive programs on health worker numbers and densities is not only a function of program scale and program effect on participating individuals, but depends also on the effect of the programs on non-participating health workers. It is plausible that participating health workers will deter non-participants from practice in underserved communities because the former will compete with the latter for patients and practice personnel. Conversely, it also seems plausible that the inflow of program participants into underserved communities attracts non-participating health workers to the same communities because the former decrease the overall work load per health worker (which may be perceived as too high) and increase opportunities for referral and exchange among colleagues. A study by Pathman and colleagues is significant insofar as it suggests "that the NHSC contributed positively to the non-NHSC primary care physician workforce in the rural underserved counties where its clinicians worked during the 1980s and 1990s" [55]. In the above discussion of summary findings from our review, we caution that the existing evidence regarding program results, effects, and impacts does not allow (strong) causal inferences. It is further important to keep in mind that the summaries are across five countries, five types of programs, programs of different geographic reach ranging from community to country, seven types of health workers, and study publication dates ranging from 1963 to 2008. Program recruitment, retention, and satisfaction outcomes differed widely, even within some of the strata defined by program location, type, geographical reach, health worker type, and time period. Health planners can use our review to gain an overview of the existing evidence. In designing future programs, however, they need to carefully consider the applicability of findings from the studies in the review to the market for health care education in their country, the specific health worker group they intend to target with a program, and the type of underserved areas they aim to supply with program participants.

## Conclusion

Financial-incentive programs for return of service are one of the few health policy interventions to improve the distribution of human resources for health on which substantial evidence exists. However, the majority of studies to date are from the US and only one study reports findings from a developing country. The existing studies show that financial-incentive programs placed substantial numbers of health workers in underserved areas and that program participants were more likely than non-participants to work in underserved areas in the long run, even though they were less likely to remain at their site of original placement. As none of the existing studies can fully rule out that the observed differences between participants and non-participants are due to selection effects, the evidence to date does not allow the inference that the programs have caused increases in the supply of health workers to underserved areas. In order to improve the scope of evidence on financial-incentive programs for return of service in underserved areas, future studies should evaluate programs from a more diverse set of countries, in particular in the developing world. In these studies, researchers should attempt to control selection biases as rigorously as possible, using selection models in observational studies and randomized controlled trials where funders and policy makers are willing to support such experiments.

## Competing interests

The authors declare that they have no competing interests.

## Authors' contributions

TB and DB jointly formulated the study design, obtained and analyzed the data, interpreted the findings, and wrote the article. Both authors read and approved the final manuscript.

## Appendix

### Endnotes

^1^In this article, unless otherwise specified, we use the term underserved area for underserved communities, regions, or populations within countries, as well as for countries where by some standards even the best-served geographic regions are underserved. The precise definition of underserved area differs across the financial-incentive programs evaluated in the studies reviewed in this article. The different definitions are reported in Additional file 1.

^2^One study evaluated jointly the NHSC and US state programs (Additional file 1). It is included in the count of both studies evaluating the NHSC and studies evaluating US state programs.

^3^Three studies reported recruitment proportions in the same program using highly overlapping samples of participants [45,59,62]. Of the three studies, we only included the one with the largest sample size in the meta-analysis [45].

^4^In the US, "Title VII grants are intended to strengthen the primary care educational infrastructure at medical schools and residency programs and to encourage physiciansin-training to pursue careers working with underserved populations" [65].

### Search algorithms

#### PubMed search

("Health Manpower" [MeSH Term] OR "Health Personnel" [MeSH Term] OR "Students" [MeSH Term] OR "Internship and Residency" [MeSH Term] OR "Education, Medical" [MeSH Term])

AND

("Medically Underserved Area" [MeSH Term] OR "Professional Practice Location" [MeSH Term] OR "Rural Health" [MeSH Term] OR "Rural Health Services" [MeSH Term] OR "Primary Health Care" [MeSH Term] OR "Family Practice" [MeSH Term] OR "Career Choice" [MeSH Term])

AND

("Financial Support" [MeSH Term] OR "Training Support" [MeSH Term] OR "Physician Incentive Plans" [MeSH Term] OR "Health Planning" [MeSH Term])

#### EMBASE search

('health care manpower'/exp OR 'health care personnel'/exp OR 'student'/exp OR 'medical education'/exp)

AND

('rural health care'/exp OR 'professional practice'/exp OR 'primary health care'/exp OR 'general practice'/exp)

AND

('student assistance program'/exp OR 'finance'/exp OR 'health care personnel management'/exp OR 'health care planning'/exp)

AND

[embase]/lim NOT [31-01-2009]/sd AND [<1950-2009]/py

#### CINAHL search

((MH "Health Manpower+") or (MH "Nursing Manpower+") or (MH "Health Personnel+") or (MH "Students+") or (MH "Internship and Residency") or (MH "Education+"))

and

((MH "Medically Underserved Area") or (MH "Rural Health") or (MH "Rural Health Services") or (MH "Primary Health Care") or (MH "Family Practice") or (MH "Career Planning and Development"))

and

((MH "Financial Support+") or (MH "Employee Incentive Programs") or (MH "Health and Welfare Planning+"))

#### NHS EED search

((Health Manpower) OR (Health Personnel) OR (Students) OR (Internship and Residency) OR (Medical Education))

AND

((Medically Underserved Area) OR (Professional Practice Location) OR (Rural Health) OR (Rural Health Services) OR (Primary Health Care) OR (Family Practice))

AND

((Career Choice) OR (Financial Support) OR (Training Support) OR (Physician Incentive Plans) OR (Health Planning)) in NHS Economic Evaluation Database

## Pre-publication history

The pre-publication history for this paper can be accessed here:



## Supplementary Material

Additional file 1 **Evaluated financial-incentive programs.** Click here for file

Additional file 2 **Reviewed studies.** Click here for file
