# Supplementary material for: Financial incentives for return of service in underserved areas: a systematic review
Source: BMC Health Serv Res. 2009 May 29;9:86. doi: 10.1186/1472-6963-9-86 (PMC2702285; doi:10.1186/1472-6963-9-86)
Supplement: Additional file 1 — Evaluated financial-incentive programs. [file 1472-6963-9-86-S1.pdf]

Additional file 1: Evaluated financial-incentive programs

Program name	Location	Period	Objective	Target group	Definition of underserved area	Financial incentive and obligation	Evaluation studies	Other references
Common-wealth Fund Medical Under-graduate Scholarship Program	Massachusetts, Mississippi, Tennessee, US	1930-1944	"[T]o alleviate medical shortages in rural areas"	Under-graduate medical students attending medical school at Tufts, Tulane, or Vanderbilt University	Rural communities with a population of 5,000 or less (subsequently raised to 10,000 or less)	*Service-requiring scholarships for medical students:*<br>Total of USD 1,300 per year over 4 years (between 1930 and 1944; amount equivalent to year-2000 USD 13,405 in 1930 and 12,719 in 1944).<br><br>In return, the students agree to spend not less than 3 years in practice in an underserved area in their state of origin.	Fitz et al. 1977 [1]	N/A
11 US state service-requiring scholarships and service-option educational loan programs	Arkansas, Georgia, Illinois, Iowa, Kentucky, Minnesota, North Carolina, North Dakota, South Carolina, Virginia, West Virginia, US	Pro-grams started between 1942 and 1968	To increase the number of physicians practicing in underserved areas	Medical students in the respective US state	Different definitions of underserved area (town with population size below a certain threshold, rural community, rural county, rural area, "area of critical need", anywhere in the state)	*Service-requiring scholarships and service-option educational loans for medical students:*<br>Between USD 1,000 and 2,775 per year for a period of two to four years (in 1970; lower amount equivalent to year-2000 USD 4,438, higher amount equivalent to year-2000 USD 10,097).	Mason 1971 [2]	N/A

Program name	Location	Period	Objective	Target group	Definition of underserved area	Financial incentive and obligation	Evaluation studies	Other references
North Carolina Rural Loan Program	North Carolina, US	Since 1945	“[T]o help smaller communities to obtain professional services”	Students of medicine, dentistry, pharmacy and nursing	“[A]ny town or village having less than 2,500 population according to the last decennial census, or area outside such towns or villages, or area approved by the Medical Care Commission that is considered to meet the spirit and intent of the student loan program”	*Educational loans with service requirements for students:*<br>USD 1,600 per year for unmarried students (in 1963; amount equivalent to year-2000 USD 9,000) and USD 1,900 for married students (in 1963; amount equivalent to year-2000 USD 10,687).<br><br>For each year the loan is received, the students agree to practice in an underserved area for one year. The loans bear an interest rate of 2%, beginning at the time the loan is advanced. The loans are repaid in monthly installments of 2% of the total amount borrowed, commencing six months after start of practice.	Bradbury 1963 [3]	N/A
Arizona Medical Student Exchange Program	Arizona, US	Since 1953	“[T]o increase the number of graduating physicians who will return to practice in Arizona”	Arizona medical students	Any area in Arizona	*Payments to medical schools to reduce students’ tuition:*<br>USD 2,000 (in 1953; amount equivalent to year-2000 USD 12,899), raised to USD 6,000 (in 1977; amount equivalent to year-2000 USD 17,050).<br><br>For each year the scholarship is received, the students agree to practice two years (1953-1957) or one year (after 1957) in Arizona.	Navin and Nichols 1977 [4]	N/A

Program name	Location	Period	Objective	Target group	Definition of underserved area	Financial incentive and obligation	Evaluation studies	Other references
Ontario Under-serviced Area Program (UAP)	Ontario, Canada	Since 1969	To increase the number of physicians per population in underserved areas in Ontario	Canadian physicians and medical students	Designation of an area as underserved area decided by a committee composed of members of the MoH, based on multiple factors: “[n]umber of doctors in the area” and “their age and health”, “type and amount of practice”, demographic profile of the population, “[s]ocioeconomic status of the area”, “[l]ocal demand for medical services”, “[a]vailability of adequate housing and office facilities for physicians”, and “[h]ealth needs and resources” [5]	*Service-requiring scholarships for medical students:*<br>CAD 5,000 per year (in 1987; amount equivalent to year-2000 USD 5,921).<br><br>For each year the scholarship is received, the students agree to spend one calendar year in general practice in an underserved area after completion of internship.<br><br>*Direct financial incentives for physicians:*<br>CAD 10,000 per year served in an underserved area for a maximum of four years (between 1980 and 1988; amount equivalent to year-2000 USD 17,591 in 1980 and year-2000 USD 11,279 in 1988).	Bass and Copeman 1975 [5];<br>Anderson and Rosenberg 1990 [6]	[7]

Program name	Location	Period	Objective	Target group	Definition of underserved area	Financial incentive and obligation	Evaluation studies	Other references
Jichi Medical University (JMU)	Japan nationwide	Since 1972	"[T]o produce rural doctors and distribute them nationwide" [8]	All physicians enrolled in JMU	Rural areas of Japan. The rural areas to which JMU graduates are sent are determined by each of Japan's 47 prefectures, "considering the demand-supply balance of physicians" [8].	*Service-requiring scholarships for medical students:*<br>Full scholarships cover "tuition, entrance and equipment fees and living expenses for the 6 years of study at JMS" [9].<br><br>In return, participants "are committed to working for medical institutions in their home prefecture for 9 years after graduation. … The 9-year obligation usually includes 3 years of post-graduate training and 6 years of rural dispatch (ie, duty)" [10].	Inoue et al. 1997 [9]; Inoue et al. 2007 [11]; Matsumoto et al. 2008 [10]; Matsumoto et al. 2008 [8]; Matsumoto et al. 2008 [12]	N/A

Program name	Location	Period	Objective	Target group	Definition of underserved area	Financial incentive and obligation	Evaluation studies	Other references
National Health Service Corps (NHSC)	US nationwide	Since 1972 (scholar-ships)<br><br>Since 1987 (loan repay-ments)	To increase the number of physicians and other health professionals in federally designated Health Professional Shortage Areas (HPSA)	Students enrolled in allopathic or osteopathic medical schools, family nurse practitioners, nurses, midwives, physician assistants, dentists	HPSA status can be assigned to areas, population groups, and facilities. HPSA status is assigned taking into account the practitioner-to-population ratio, availability and accessibility of clinicians in adjacent areas, indicators of need (such as infant mortality) and capacity (such as poverty levels) [13]	*Service-requiring scholarships for students:*<br>Full scholarships covering tuition, fees, and "other reasonable educational expenses, such as books, supplies, and equipment" [13].<br><br>For each year the scholarship is received, the students agree to serve one year at a location designated as HPSA, with a minimum commitment of two years.<br><br>*Loan repayment programs for physicians:*<br>Maximum repayment of USD 25,000 per year for a required initial two-year contract (in 2007; amount equivalent to year-2000 USD 20,336). One year amendments for a maximum of USD 35,000 per year (in 2008; amount equivalent to year-2000 USD 28,470).	Woolf et al. 1981 [14]; Stamps and Kuriger 1983 [15]; Brown et al. 1990 [16]; Stone et al. 1991 [17]; Pathman et al. 1992 [18]; Pathman et al. 1994 [19]; Pathman et al. 1994 [20]; Pathman and Konrad 1996 [21]; Rosenblatt et al. 1996 [22]; Cullen et al. 1997 [23]; Xu et al. 1997 [24]; Xu et al. 1997 [25]; Singer et al. 1998 [26]; Rabinowitz et al. 2000 [27]; Rabinowitz et al. 2001 [28]; Mofidi et al. 2002 [29];	[13, 30]

Program name	Location	Period	Objective	Target group	Definition of underserved area	Financial incentive and obligation	Evaluation studies	Other references
							Brooks et al. 2003 [31]; Porterfield et al. 2003 [32]; Probst et al. 2003 [33]; Holmes 2004 [34]; Holmes 2005 [35]; Pathman et al. 2005 [36]; Pathman et al. 2006 [37]; Rittenhouse et al. 2008 [38]	
Scholarship for Indian students in health sciences	Arizona, Utah, Colorado, New Mexico, US	Since 1972	To supply health manpower to the Navajo Indian Reservations and immediately adjacent communities	Indian medical students	Navajo Indian Reservations and immediately adjacent communities	*Service-requiring scholarships for medical students:* Ranging from USD 650 to 11,000 per year (in 1980; lower amount equivalent to year-2000 USD 1,358 and higher amount equivalent to year-2000 USD 22,988).	Weiss et al. 1980 [39]	N/A

Program name	Location	Period	Objective	Target group	Definition of underserved area	Financial incentive and obligation	Evaluation studies	Other references
Oklahoma Rural Medical Education Scholarship Loan	Oklahoma, US	Since 1975	“[T]o increase the number of practicing physicians in underserved and rural areas” (Holmes and Miller 1985)	Osteopathic and allopathic medical students	Rural communities in Oklahoma	*Service-requiring scholarships for medical students:*<br>USD 15,500 per year over four years (in 2008; amount equivalent to year-2000 USD 12,202).<br><br>For each year the scholarship is received, the students agree to practice one year in a rural community in Oklahoma (after residency in a primary care specialty).	Holmes and Miller 1985 [40];<br>Lapolla et al. 2004 [41]	[42]
NHSC<br><br>Indian Health Service Corps<br><br>US state service-requiring scholarships<br><br>State loan repayment programs<br><br>Practice and hospital-sponsored direct financial incentives	US nationwide	Pro-grams opera-ting in the 1980s and 1990s	To increase the number of physicians in underserved areas	Medical students, medical residents, and physicians	Different definitions of underserved area	*Service-requiring scholarships, loan repayment programs, direct financial incentives:*<br>NHSC: See above.<br><br>Indian Health Service Corps: Up to USD 20,000 per year (in 2006; amount equivalent to year-2000 USD 17,083).<br><br>For each year the scholarship is received, the students agree to serve one year in an Indian health program, with a minimum commitment of two years [43].<br><br>State scholarships: See above and below for examples.<br><br>State loan repayment programs: See above and below for examples.<br><br>Practice and hospital-sponsored direct financial incentives: Information on size of incentive and obligation not available.	Pathman et al. 2000 [44]	[43]

Program name	Location	Period	Objective	Target group	Definition of underserved area	Financial incentive and obligation	Evaluation studies	Other references
New South Wales Department of Health Rural Resident Medical Officer Program (Cadetship Program)	New South Wales (NSW), Australia	Since 1989	"[T]o help overcome a junior doctor workforce shortage in rural hospitals" in New South Wales<br><br>"[T]o increase recruitment to the rural medical workforce" (Dunabin et al. 2006)	Australian and New Zealand medical students	Rural hospitals in the NSW Rural Hospital Network	*Service-requiring scholarships for medical students:*<br>AUD 15,000 per year over the two final years of medical school (in 2007; amount equivalent to year-2000 USD 12,458).<br><br>In return, the students agree to serve two of their first three postgraduate years in a rural hospital belonging to the NSW Rural Hospital Network.	Dunbabin et al. 2006 [45]	[46]

Program name	Location	Period	Objective	Target group	Definition of underserved area	Financial incentive and obligation	Evaluation studies	Other references
Community Scholarship Program (CSP)<br><br>Health Sciences Scholarship Program (HSSP)<br><br>Recruitment and Retention Community Program (RRCP)<br><br>State Loan Repayment Program (SLRP)	West Virginia, US	CSP: 1991-1997<br><br>HSSP: Since 1996	"[T]o attract medical students, residents, and physicians to practice in rural and underserved areas" of West Virginia	CSP: medical students from HPSA<br><br>HSSP: fourth year medical students<br><br>RRCP: medical residents, physicians and other qualified health profes-sionals<br><br>SLRP: physicians and other qualified health profes-sionals	HPSA	*CSP: service-requiring scholarships for medical students:*<br>Amount determined by HPSA community who co-sponsors the scholarship (with additional funding from federal and state funds).<br><br>For each year the scholarship is received, the students agree to serve one year in the HPSA where their home is located.<br><br>*HSSP: service-requiring scholarships for medical students:*<br>USD 10,000 (in 2001; amount equivalent to year-2000 USD 9,725)<br><br>For the award, the students agree to serve two years in an underserved area.<br><br>*RRCP: direct financial incentives to medical residents, physicians, and other qualified health personnel:*<br>Maximum of USD 20,000 per year for up to six years (in 2001; amount equivalent to year-2000 USD 19,450)<br><br>For each year the award is received, the recipients agree to serve one year in an underserved area.<br><br>*SLRP: direct financial incentives to physicians and other qualified health professionals:*<br>Maximum of USD 40,000 (in 2001; amount equivalent to year-2000 USD 38,901) for a commitment to serve two years at a non-profit site in a HPSA. The award can be received twice.	Jackson et al. 2003 [47]	N/A

Program name	Location	Period	Objective	Target group	Definition of underserved area	Financial incentive and obligation	Evaluation studies	Other references
20 US state service-requiring scholarship programs<br><br>12 state service-option educational loan programs<br><br>24 state loan repayment programs<br><br>6 state direct financial-incentive programs for medial residents<br><br>7 state direct financial-incentive programs for fully trained health professionals	40 US states	All US state pro-grams opera-ting in 1996	"[T]o entice young generalist physicians into rural and medically underserved areas"	Medical students, medical residents, and physicians	Different definitions of underserved area	Across all programs on average USD 14,000 per year of service (in 1996; amount equivalent to year-2000 USD 15,365) (differences between award means of the five program types not significant, $p = 0.55$).<br><br>*Service-requiring scholarships:*<br>For each year the scholarship is received, the students agree to serve one year in an underserved area.<br><br>*Service-option educational loans:*<br>The medical students can either repay the loan at standard interest rates or repay the loan by serving one year in an underserved area per year of receipt of loan.<br><br>*Loan repayment programs:*<br>Medical residents commit to service in an underserved area in exchange for loan repayment (commitment usually near the end of residency training).<br><br>*Direct financial incentives for medical residents:*<br>Medical residents commit to service in an underserved area in exchange for the monetary reward (commitment usually at the beginning of the residency).<br><br>*Direct financial incentives for fully trained health professionals:*<br>Medical residents commit to service in an underserved area in exchange for the monetary reward (commitment usually near the end of residency training).	Pathman et al. 2004 [48]	N/A

Program name	Location	Period	Objective	Target group	Definition of underserved area	Financial incentive and obligation	Evaluation studies	Other references
Friends of Mosvold Scholarship Scheme (FOMSS)	Umkhan-yakude district, KwaZulu-Natal, South Africa	Since 1998	To "help integrate graduates into the workforce in the district"	Students from Umkhan-yakude district who have been admitted to a tertiary health care education institution, completed at least 2 weeks of work experience at one of the hospitals in the district, and were selected by a committee of local residents	District of Umkhanyakude	*Service-requiring scholarships for students admitted to a tertiary health care education institution:*<br>"Funds for university tuition, books, residence fees and food" [49].<br><br>For each year the scholarship is received, the students agree to work one year as health professionals in Umkhanyakude district.	Ross 2007 [50]	[49]

USD = United States dollar, CAD = Canadian dollar, AUD = Australian dollar, MoH = Ministry of Health, N/A = not applicable.

References

1. Fitz RH, Mawardi BH, Wilber J: **Scholarships for rural medicine. The Commonwealth Fund experience with a pre-World War II indenture program**. *Trans Am Clin Climatol Assoc* 1977, **88**:191-196.
2. Mason HR: **Effectiveness of student aid programs tied to a service commitment**. *J Med Educ* 1971, **46**(7):575-583.
3. Bradbury SF: **The North Carolina Medical Care Commission: evaluation of the Rural Loan Program by recipients of medical and dental loans**. *N C Med J* 1963, **24**:489-491.
4. Navin TR, Nichols AW: **Evaluation of the Arizona Medical Student Exchange Program**. *J Med Educ* 1977, **52**(10):817-823.
5. Bass M, Copeman WJ: **An Ontario solution to medically underserviced areas: evaluation of an ongoing program**. *Canadian Medical Journal* 1975, **113**:403-407.
6. Anderson M, Rosenberg MW: **Ontario's underserviced area program revisited: an indirect analysis**. *Soc Sci Med* 1990, **30**(1):35-44.
7. Copeman WJ: **The underserviced area program of the Ministry of Health of Ontario**. *Canadian Family Physician* 1987, **33**:1683-1685.
8. Matsumoto M, Inoue K, Kajii E: **Characteristics of medical students with rural origin: implications for selective admission policies**. *Health Policy* 2008, **87**(2):194-202.
9. Inoue K, Hirayama Y, Igarashi M: **A medical school for rural areas**. *Med Educ* 1997, **31**(6):430-434.
10. Matsumoto M, Inoue K, Kajii E: **A contract-based training system for rural physicians: follow-up of Jichi Medical University graduates (1978-2006)**. *J Rural Health* 2008, **24**(4):360-368.
11. Inoue K, Matsumoto M, Sawada T: **Evaluation of a medical school for rural doctors**. *J Rural Health* 2007, **23**(2):183-187.
12. Matsumoto M, Inoue K, Kajii E: **Long-term effect of the home prefecture recruiting scheme of Jichi Medical University, Japan**. *Rural Remote Health* 2008, **8**(3):930.
13. **About NHSC** [http://nhsc.bhpr.hrsa.gov/about/]
14. Woolf MA, Uchill VL, Jacoby I: **Demographic factors associated with physician staffing in rural areas: the experience of the National Health Service Corps**. *Med Care* 1981, **19**(4):444-451.
15. Stamps PL, Kuriger FH: **Location decisions of National Health Service Corps physicians**. *Am J Public Health* 1983, **73**(8):906-908.
16. Brown J, Stone V, Sidel VW: **Decline in NHSC physicians threatens patient care**. *Am J Public Health* 1990, **80**(11):1395-1396.
17. Stone VE, Brown J, Sidel VW: **Decreasing the field strength of the National Health Service Corps: will access to care suffer?** *J Health Care Poor Underserved* 1991, **2**(3):347-358.
18. Pathman DE, Konrad TR, Ricketts TC, 3rd: **The comparative retention of National Health Service Corps and other rural physicians. Results of a 9-year follow-up study**. *JAMA* 1992, **268**(12):1552-1558.
19. Pathman DE, Konrad TR, Ricketts TC, 3rd: **Medical education and the retention of rural physicians**. *Health Serv Res* 1994, **29**(1):39-58.

20. Pathman DE, Konrad TR, Ricketts TC, 3rd: **The National Health Service Corps experience for rural physicians in the late 1980s**. *JAMA* 1994, **272**(17):1341-1348.
21. Pathman DE, Konrad TR: **Minority physicians serving in rural National Health Service Corps sites**. *Med Care* 1996, **34**(5):439-454.
22. Rosenblatt RA, Saunders G, Shreffler J, Pirani MJ, Larson EH, Hart LG: **Beyond retention: National Health Service Corps participation and subsequent practice locations of a cohort of rural family physicians**. *J Am Board Fam Pract* 1996, **9**(1):23-30.
23. Cullen TJ, Hart LG, Whitcomb ME, Rosenblatt RA: **The National Health Service Corps: rural physician service and retention**. *J Am Board Fam Pract* 1997, **10**(4):272-279.
24. Xu G, Veloski JJ, Hojat M, Politzer RM, Rabinowitz HK, Rattner S: **Factors influencing physicians' choices to practice in inner-city or rural areas**. *Acad Med* 1997, **72**(12):1026.
25. Xu G, Fields SK, Laine C, Veloski JJ, Barzansky B, Martini CJ: **The relationship between the race/ethnicity of generalist physicians and their care for underserved populations**. *Am J Public Health* 1997, **87**(5):817-822.
26. Singer JD, Davidson SM, Graham S, Davidson HS: **Physician retention in community and migrant health centers: who stays and for how long?** *Med Care* 1998, **36**(8):1198-1213.
27. Rabinowitz HK, Diamond JJ, Veloski JJ, Gayle JA: **The impact of multiple predictors on generalist physicians' care of underserved populations**. *Am J Public Health* 2000, **90**(8):1225-1228.
28. Rabinowitz HK, Diamond JJ, Markham FW, Paynter NP: **Critical factors for designing programs to increase the supply and retention of rural primary care physicians**. *JAMA* 2001, **286**(9):1041-1048.
29. Mofidi M, Konrad TR, Porterfield DS, Niska R, Wells B: **Provision of care to the underserved populations by National Health Service Corps alumni dentists**. *J Public Health Dent* 2002, **62**(2):102-108.
30. Politzer RM, Trible LQ, Robinson TD, Heard D, Weaver DL, Reig SM, Gaston M: **The National Health Service Corps for the 21st century**. *J Ambul Care Manage* 2000, **23**(3):70-85.
31. Brooks RG, Mardon R, Clawson A: **The rural physician workforce in Florida: a survey of US- and foreign-born primary care physicians**. *J Rural Health* 2003, **19**(4):484-491.
32. Porterfield DS, Konrad TR, Porter CQ, Leysieffer K, Martinez RM, Niska R, Wells B, Potter F: **Caring for the underserved: current practice of alumni of the National Health Service Corps**. *J Health Care Poor Underserved* 2003, **14**(2):256-271.
33. Probst JC, Samuels ME, Shaw TV, Hart GL, Daly C: **The National Health Service Corps and Medicaid inpatient care: experience in a southern state**. *South Med J* 2003, **96**(8):775-783.
34. Holmes GM: **Does the National Health Service Corps improve physician supply in underserved locations?** *Eastern Economic Journal* 2004, **30**(4):563-581.
35. Holmes GM: **Increasing physician supply in medically underserved areas**. *Labour Economics* 2005, **12**:697-725.

36. Pathman DE, Fryer GE, Green LA, Phillips RL: **Changes in age-adjusted mortality rates and disparities for rural physician shortage areas staffed by the National Health Service Corps: 1984-1998**. *J Rural Health* 2005, **21**(3):214-220.
37. Pathman DE, Fryer GE, Jr., Phillips RL, Smucny J, Miyoshi T, Green LA: **National Health Service Corps staffing and the growth of the local rural non-NHSC primary care physician workforce**. *J Rural Health* 2006, **22**(4):285-293.
38. Rittenhouse DR, Fryer GE, Jr., Phillips RL, Jr., Miyoshi T, Nielsen C, Goodman DC, Grumbach K: **Impact of Title VII training programs on community health center staffing and National Health Service Corps participation**. *Ann Fam Med* 2008, **6**(5):397-405.
39. Weiss LD, Wiese WH, Goodman AB: **Scholarship support for Indian students in the health sciences: an alternative method to address shortages in the underserved area**. *Public Health Rep* 1980, **95**(3):243-246.
40. Holmes JE, Miller DA: **A study of 138 return service scholarship applications awarded by the Oklahoma Physician Manpower Training Commission**. *J Okla State Med Assoc* 1985, **78**(10):384-388.
41. Lapolla M, Brandt EN, Jr., Barker A, Ryan L: **State public policy: the impacts of Oklahoma's physician incentive programs**. *J Okla State Med Assoc* 2004, **97**(5):190-194.
42. **Rural medical education scholarship loan program** [http://www.pmtc.state.ok.us/ruraled.htm]
43. Indian Health Service: *Loan repayment program*. Rockville, MD: Indian Health Service; 2006.
44. Pathman DE, Konrad TR, King TS, Spaulding C, Taylor DH: **Medical training debt and service commitments: the rural consequences**. *J Rural Health* 2000, **16**(3):264-272.
45. Dunbabin JS, McEwin K, Cameron I: **Postgraduate medical placements in rural areas: their impact on the rural medical workforce**. *Rural Remote Health* 2006, **6**(2):481.
46. **NSW rural resident medical officer cadetship** [http://www.nswrdn.com.au/site/index.cfm?module=RDNSCHOLARSHIPS&leca=82&pagemode=indiv&page_id=1839]
47. Jackson J, Shannon CK, Pathman DE, Mason E, Nemitz JW: **A comparative assessment of West Virginia's financial incentive programs for rural physicians**. *J Rural Health* 2003, **19 Suppl**:329-339.
48. Pathman DE, Konrad TR, King TS, Taylor DH, Jr., Koch GG: **Outcomes of states' scholarship, loan repayment, and related programs for physicians**. *Med Care* 2004, **42**(6):560-568.
49. Ross AJ, Couper ID: **Rural scholarship schemes: a solution to the human resource crisis in rural district hospitals?** *South African Family Practice* 2004, **46**(1):5-6.
50. Ross AJ: **Success of a scholarship scheme for rural students**. *S Afr Med J* 2007, **97**(11):1087-1090.
