# Supplementary material for: Financial incentives for return of service in underserved areas: a systematic review
Source: BMC Health Serv Res. 2009 May 29;9:86. doi: 10.1186/1472-6963-9-86 (PMC2702285; doi:10.1186/1472-6963-9-86)
Supplement: Additional file 2 — Reviewed studies. [file 1472-6963-9-86-S2.pdf]

Additional file 2: Reviewed studies

Program name	Study	Study outcomes	Study design	Sample criteria and sample size	Data sources	Outcome measures and study findings	Conclusions	Methodological limitations
Common-wealth Fund Medical Under-graduate Scholarship Program	Fitz et al. 1977 [1]	*Program results:* Recruitment<br><br>Retention (in any underserved area)	Description of program outcomes	All individuals who ever took part in the program (N = 144)	Common-wealth Fund records	*Proportion of participants who completed their practice obligation in 1973 or before:*<br>Of 144 participants, 11 (8%) did not graduate from medical school or died before completing the obligation.<br><br>Of 133 participants available for practice, 74 (54%) completed the obligation and 5 (4%) repaid the financial incentive, while the remainder defaulted.<br><br>*Proportion of participants who practiced in small communities in 1973 (43 years after program start and 29 years after program cessation):*<br>Of 99 former recipients who were still in practice in 1973, 50 (51%) practiced in communities of less than 25,000 population.	Almost half of all participants did not fulfill their obligation to practice in an underserved area.<br><br>However, it is difficult to evaluate the program because of WWII. Most of the participants who did not complete their obligation (52) requested and obtained release after WWII.<br><br>Nevertheless, about half of the participants practiced in small communities for most of their working lives.	Descriptive study<br><br>No control group<br><br>Duration of individual retention not taken into account<br><br>WWII created an exceptional situation during program operation.

Program name	Study	Study outcomes	Study design	Sample criteria and sample size	Data sources	Outcome measures and study findings	Conclusions	Methodological limitations
11 US state service-requiring scholarships and service-option educational loan programs	Mason 1971 [2]	*Program results:* Recruitment<br><br>Retention (in any underserved area)	Description of program outcomes	All individuals who ever participated in one of the state programs and were available for practice in 1970<br>(N = 1,089)	Records of the individual state programs	*Proportion of participants who started serving or completed their practice obligation in 1970 or before:*<br>Of 1,089 participants available for practice, 658 (60%) started serving or completed the obligation and 406 (37%) repaid the financial incentive, while the remainder defaulted.<br><br>*Proportion of participants who remained in a rural community in their state after completion of their practice obligation (neither date of measurement nor duration information provided):*<br>Georgia: 50%<br>Kentucky: 90%<br>North Carolina: 65%	Two fifths of participants did not fulfill their obligation to practice in an underserved area.<br><br>The proportion of participants recruited to rural areas varied widely across programs.<br><br>Between 50% and 90% of participants remained in rural communities after completion of their obligation.	Descriptive study<br><br>No control group<br><br>Duration of individual retention not taken into account

Program name	Study	Study outcomes	Study design	Sample criteria and sample size	Data sources	Outcome measures and study findings	Conclusions	Methodological limitations
North Carolina Rural Loan Program	Bradbury 1963 [3]	*Program results:* Recruitment<br><br>Retention (in the same underserved area)<br><br>Participant satisfaction	Description of program outcomes	All students who were ever enrolled in the program between 1945 and 1963 (N = 320)	Records of the North Carolina Medical Care Commission	*Proportion of participants who started serving or completed their practice obligation in 1963 or before:* Of 320 participants, 120 (38%) were still in school, post-graduate training or the military, 46 (14%) withdrew from school or failed academically, and 13 (4%) withdrew their application to participate in the program or died.<br><br>Of 141 participants available for practice, 106 (75%) started serving or completed the obligation and 35 (25%) defaulted on the obligation.<br><br>*Proportion of participants who intended to leave the community after completion of their obligation:* Of 36 respondents, 29 (81%) stated that they intended to remain in the community, while 6 (17%) planned further training and one planned to move to an urban area.	A quarter of participants did not fulfill their obligation to practice in an underserved area.<br><br>71% of participants in the financial-incentive program were satisfied with their overall experience.	Descriptive study<br><br>No control group<br><br>Study of retention investigates only the intention to leave the practice location and not the actual location decision.

Program name	Study	Study outcomes	Study design	Sample criteria and sample size	Data sources	Outcome measures and study findings	Conclusions	Methodological limitations
						Proportion of participants who found the loan amount sufficient: Of 38 respondents who provided a valid answer to the question "Did you find the amount of the loan sufficient together with your resources to alleviate any undue concern over financial problems during the time you were in school?", 29 (76%) answered "yes", while the remainder answered "no".<br><br>*Proportion of participants who would participate again in the same program:* Of 38 respondents who provided a valid answer to the question whether they would participate again in the financial-incentive program, 27 (71%) answered "yes", while 11 (29%) answered "no".		

Program name	Study	Study outcomes	Study design	Sample criteria and sample size	Data sources	Outcome measures and study findings	Conclusions	Methodological limitations
Arizona Medical Student Exchange Program	Navin and Nichols 1977 [4]	*Program results:* Recruitment<br><br>Retention (in any underserved area)<br><br>*Program impacts:* Health system	Description of program outcomes<br><br>Time series	All students who ever participated in the program between 1953 and 1977 and who completed their medical training in 1975 or before (N = 149)	Records of the Western Interstate Commission for Higher Education	*Proportion of participants who had started serving or completed their practice obligation in 1975 or before:* Of 149 participants, 67 (45%) served the obligation in a metropolitan area within Arizona, 21 (14%) served the obligation in a non-metropolitan area in Arizona, and 55 (37%) repaid the financial incentive, while the remainder defaulted.<br><br>*Proportion of participants who remained in rural communities of their state after completion of their practice obligation:* >85%<br><br>*Time series of medical student density in Arizona:* The per-capita number of medical students did not increase from 1953 to 1967 (consistently 20% below national average), but increased steeply from 1968 onwards.	About two fifths of participants did not fulfill their obligation to practice in an underserved area.<br><br>85% of participants who completed their obligation remained in Arizona.<br><br>The program did not succeed in increasing the medical student population density in Arizona. The steep increase in per-capita medical students in 1968 is attributed to the opening of the first medical school in Arizona in that year.	*Program outcome:* Descriptive study<br><br>No control group<br><br>Duration of individual retention not taken into account<br><br>*Program impact:* No analysis of time series undertaken except for visual impression<br><br>No control for confounding by other variables that changed over time

Program name	Study	Study outcomes	Study design	Sample criteria and sample size	Data sources	Outcome measures and study findings	Conclusions	Methodological limitations
Ontario Under-serviced Area Program (UAP)	Bass and Copeman 1975 [5]	*Program results:* Recruitment<br>Retention (in the same underserved area)<br>*Program impacts:* Health system	Description of program outcomes<br>Time series	All participating students who completed their internship in 1974 or before (N = 104)<br>7 annual values (1966-1972) of the number of physicians in each of three geographical areas (all Ontario, northern Ontario, communities in northern Ontario with population of less than 15,000)	Canadian Medical Directory	*Proportion of participating medical students who started serving or completed their practice obligation in 1974 or before:*<br>Of 104 students, 55 (53%) started serving or completed the obligation and 49 (47%) repaid the financial incentive.<br>*Proportion of students who in 1974 remained in the original placement location after completion of their practice obligation:*<br>74%<br>*Time series of total number of physicians (expressed relative to their 1966 baseline value):*<br>From 1966 to 1972 the relative number of physicians increased monotonically in all Ontario (from 1.0 to over 1.3) and in northern Ontario (from 1.0 to almost 1.2).<br>In communities in northern Ontario with a population of less than 15,000 the relative number of physicians declined slightly from 1966 to 1969 (i.e., before the program was introduced), while it increased steeply from 1970 (after introduction of the program) to 1972 (from 1.0 to almost 1.3).	About half of participants did not fulfill their obligation to practice in an underserved area.<br>About three quarters of participants who completed their obligation remained at the original placement location.<br>The time series suggests that the program was effective in increasing the number of physicians practicing in small communities in northern Ontario.	*Program outcome:* Descriptive study<br>No control group<br>Duration of individual retention not taken into account<br>*Program impact:* No analysis of time series undertaken except for visual impression<br>No control for confounding by other variables that changed over time<br>Ecological bias possible (because units of observation are groups of communities)

Program name	Study	Study outcomes	Study design	Sample criteria and sample size	Data sources	Outcome measures and study findings	Conclusions	Methodological limitations
UAP	Anderson and Rosenberg 1990 [6]	*Program impacts:* Health system	Before-after comparison of physician density in northern counties of Ontario (where most underserved areas are located) vs. in Ontario overall over a 30- year period (1956-1986, i.e., covering time before and after introduction of UAP in 1969)	Panel of all 10 counties in northern Ontario observed at seven points in time	Canadian Medical Directory<br>Census Canada	*Physician population density in 1986 relative to physician population density in 1956:* 1.86-4.88 across the 10 northern counties<br>*Location quotient (physician density in the counties of northern Ontario relative to the physician population density in Ontario as a whole) in 1986 relative to location quotient in 1956:* 0.88-1.33 across the 10 northern counties	The fact that the location quotient improved little over the 30-year observation period suggests that the increase in physician population density in northern Ontario (where most of the underserved areas in Ontario were located) was caused by an overall increase in physicians in the state rather than by UAP.	Observational study<br>No control for confounding by other variables that changed over time<br>Ecological bias possible (because not all communities in one county are underserved)

Program name	Study	Study outcomes	Study design	Sample criteria and sample size	Data sources	Outcome measures and study findings	Conclusions	Methodological limitations
Jichi Medical University (JMU)	Inoue et al. 1997 [7]	*Program results:* Recruitment<br>Retention (in the same underserved area)<br>Retention (in any underserved area)	Description of program outcomes	All physicians who graduated from JMU in one of the first eighteen graduation cohorts of the university (N = 1,871)	Mail survey of JMU graduates in 1995	*Proportion of participants who "observed the contract in compliance with the conditions for receiving financial aid" in 1995 or before:* Of 1,871 participants, 1,796 (96%), observed the contract, while 75 (4%) "repaid the loans to dissolve the contract requiring them to complete 9 years of medical employment in a rural area".<br>*Proportion of participants who in 1995 were still practicing in the prefecture of original placement after completion of their practice obligation:* Of 924 participants who completed the obligation in 1995 or before, 620 (67%) were still practicing in the prefecture of original placement.<br>*Proportion of participants who in 1995 were still practicing in a rural area after completion of their practice obligation:* Of 924 participants who completed the obligation in 1995 or before, 305 (33%) were still practicing in a rural area.	96% of all participants fulfilled their obligation to practice in an underserved area.<br>About two thirds of participants remained in the prefecture of original placement after completion of their obligation.<br>A substantial proportion of participants left rural practice after completion of their obligation.	Descriptive study<br>No control group<br>Duration of individual retention not taken into account

Program name	Study	Study outcomes	Study design	Sample criteria and sample size	Data sources	Outcome measures and study findings	Conclusions	Methodological limitations
JMU	Inoue et al. 2007 [8]	*Program results:* Recruitment<br>*Program effects:* Provision of care (in any underserved area)	Description of program outcomes	All JMU participants (N = 1,661)	Japanese National Physician Census	*Proportion of participants who started serving or completed their rural practice obligation in 1994 or before:* "Only 2% of JMS [Jichi Medical School] did not observe the rural practice obligation" [8].<br>*Proportion of participants of all physicians in Japan vs. proportion of participants in rural areas:* Participants "accounted for only 0.7% of all the physicians in Japan. However, they accounted for 4.2%, 1.5%, 1.8%, and 3.0% of the physicians in small population, remote, mountain, and medically underserved municipalities, respectively" [8].	98% of participants fulfilled their obligation to practice in an underserved area.<br>Participants were more likely than non-participants to practice in a rural area.	Descriptive study<br>Observational sub-study without primary data extraction<br>Selection bias due to selective participation in the JMU not controlled for
JMU	Matsumoto et al. 2008 [9]	*Program effects:* Provision of care (in any underserved area)	Retrospective cohort study	All JMU graduates in 1994 (N = 1,635) and 2004 (N = 2,641)<br>All non-JMU physicians in 1994 (N = 228,825) and 2004 (N = 260,041)	Mail surveys of JMU graduates. The mail surveys were, followed by telephone surveys of those participants who did not respond to the mail survey.<br>Japanese National Physician Census	*Proportion of physicians who practiced in any rural area: all JMU graduates vs. JMU graduates after completion of their practice obligation vs. non-JMU graduates:* 21% vs. 13% vs. 3% (in 1994)<br>15% vs. 11% vs. 3% (in 2004)	After having completed their obligation to practice in an underserved area JMU graduates were about four times more likely to work in rural areas than non-JMU graduates.	Observational study<br>Selection bias due to selective participation in the JMU not controlled for

Program name	Study	Study outcomes	Study design	Sample criteria and sample size	Data sources	Outcome measures and study findings	Conclusions	Methodological limitations
JMU	Matsumoto et al. 2008 [10]	*Program results:* Retention (in the same underserved area)<br>Retention (in any underserved area)	Retrospective study	All JMU graduates who matriculated since 1972 and completed their 9-year practice obligation in 2006 or before (N = 1,929)	Mail surveys in 2000, 2004, and 2006. The mail surveys were, followed by telephone surveys of those participants who did not respond to the mail survey.	*Proportion of JMU graduates who practiced in the prefecture of original placement after completion of their practice obligation: graduates of rural background vs. graduates of urban background:* 74% vs. 75% (p<0.76)<br>*Proportion of JMU graduates who practiced in any rural area after completion of their practice obligation: graduates of rural background vs. graduates of urban background:* 21% vs. 12% (p<0.001)<br>*Odds ratio of retention in the prefecture of original placement: at first practice site: graduates of rural background vs. graduates of urban background:* aOR = 0.77 (p = 0.16), when controlling for sex, age at entrance into JMU, years after graduation, type of high school, parental academic background, and change of academic standing throughout undergraduate medical training	Retention in the prefecture of original placement was high and did not differ significantly by geographical background of participants. Large proportions of participants left rural practice after completion.<br>Holding other factors constant, retention in rural areas was about twice as high in participants of rural background than in participants of urban background.	Observational study

Program name	Study	Study outcomes	Study design	Sample criteria and sample size	Data sources	Outcome measures and study findings	Conclusions	Methodological limitations
						Odds ratio of retention in any rural area: graduates of rural background vs. graduates of urban background:<br>aOR = 1.98 (p = 0.001), when controlling for sex, age at entrance into JMU, years after graduation, type of high school, parental academic background, and change of academic standing throughout undergraduate medical training		
JMU	Matsumoto et al. 2008 [11]	*Program results:* Recruitment<br><br>Retention (in the same underserved area)	Description of program outcomes	All participants who had graduated from JMU by 1991 (N = 1,477)	Mail surveys in 2000, 2004, and 2006. The mail surveys were, followed by telephone surveys of those participants who did not respond to the mail survey.	*Proportion of participants who completed their rural practice obligation in 2000 or before:*<br>Of 1,477 participants, 1,255 (85%) completed the obligation, 127 (9%) were still under contract due to contract extension, 69 (5%) dissolved the contract, and 26 (2%) could not be contacted.<br><br>*Proportion of participants who completed their practice obligation in 2000 or before and thereafter remained in the prefecture of original placement for at least 6 years:*<br>70%	95% of participants fulfilled their obligation to practice in an underserved area.<br><br>Retention in the prefecture of original placement was high.	Descriptive study<br><br>No control group

Program name	Study	Study outcomes	Study design	Sample criteria and sample size	Data sources	Outcome measures and study findings	Conclusions	Methodological limitations
National Health Service Corps (NHSC)	Woolf et al. 1981 [12]	*Program impacts:* Health system	Univariate comparison of means of demographic, economic, health, and education variables between the two types of sites<br><br>Discriminant analysis	All communities that were eligible to receive a NHSC physician and were continuously staffed from October 1975 to October 1976 (N = 76)<br><br>All communities that were eligible to receive a NHSC physician before August 1975 and had never been staffed up to August 1977 (N = 78)	NHSC records<br><br>Health Resources and Services Administration Area Resource File	*Means comparison:* Staffed communities had significantly higher median family income, lower poverty prevalence, higher income growth, lower infant mortality, lower unemployment, and higher median educational attainment.<br><br>*Discriminant analysis:* Seven variables contribute significantly and substantially to separation given the other variables in the discriminant function (sign of coefficient in parentheses): income growth (-), poverty prevalence (-), physician population density (-), employment ratio (+), infant mortality rate (-), median family income (+), proportion of people 65 years of age or older (-).	Underserved communities that were economically worse-off and had worse population health were less likely to receive a program participant than underserved communities that were economically better-off and had better population health.	Observational study<br><br>Study covers only the first few years of the NHSC program.<br><br>Ecological bias possible (because community characteristics are measured at the level of the county)
NHSC	Stamps and Kuriger 1983 [13]	*Program results:* Retention (in any underserved area)	Descriptive study	All NHSC physicians practicing in New England states, New York, Pennsylvania, Maryland and Virginia at the time of the survey (N = 100)	Mail survey	*Proportion of NHSC physicians who intend to practice in a rural area after completion of their practice obligation:* Of 100 physicians, 56 (56%) expressed intention to practice in a rural area, 15 (15%) were uncertain, and the remainder did not intend to practice in a rural area.	More than half of participants who were currently fulfilling their obligation intended to practice in a rural area after completing their obligation.	Descriptive study<br><br>Study of retention investigates only the intention to practice in a rural area and not the actual location decision.

Program name	Study	Study outcomes	Study design	Sample criteria and sample size	Data sources	Outcome measures and study findings	Conclusions	Methodological limitations
NHSC	Stone et al. 1991 [14] and Brown et al. 1990 [15]	*Program results:* Retention (in the same underserved area)<br><br>Participant satisfaction<br><br>Family satisfaction	Descriptive study	All NHSC physicians completing their practice obligation in 1989<br>(N = 401)	Mail survey	*Proportion of participants who intended to leave NHSC practice location after completion of their practice obligation:*<br>Of 397 respondents who provided valid information on their intention to leave their NHSC practice location, 265 (67%) stated that they intended to leave.<br><br>*Proportion of participants who intended to leave their NHSC practice location who provided the following reasons for leaving:*<br>"The most commonly cited reasons for leaving (each respondent could give two primary reasons) were: 1) the site was geographically isolated or was unpleasant in some other way (61 percent); 2) salary at the NHSC site was insufficient (31.5 percent); 3) on-call and clinical responsibilities associated with the NHSC position were excessive (28.5%); and 4) children's needs or spouse's career or other needs were unmet (26.2%)" [14].	Only about one third of participants who were currently fulfilling their practice obligation intended to remain in their placement practice after completing their obligation.<br><br>The major reasons for intending to leave the placement site were dissatisfaction with the community, the salary, and the workload, as well as unmet needs of family members.	Descriptive study<br><br>No control group<br><br>Study of retention investigates only the intention to leave the practice location and not the actual location decision.

Program name	Study	Study outcomes	Study design	Sample criteria and sample size	Data sources	Outcome measures and study findings	Conclusions	Methodological limitations
NHSC	Pathman et al. 1992 [16]	*Program effects:* Retention (in the same underserved area)<br><br>Retention (in any underserved area)	Retrospective cohort study	Primary care physicians practicing in a rural county who were selected in a national stratified sample in 1981, were still alive in 1990, could be contacted and responded to a mail survey in 1990<br>(N = 304)	Mail survey conducted by the Cecil G. Sheps Center for Health Services Research, University of North Carolina at Chapel Hill	*Hazard ratio of non-retention in the same practice as in 1981: NHSC vs. non-NHSC physicians:*<br>2.11 ($p < 0.0001$)<br><br>1.98 ($p = 0.0002$), when controlling for training in internal medicine and stated importance of small community living<br><br>*Hazard ratio of non-retention in any rural practice: NHSC vs. non-NHSC physicians:*<br>1.74 ($p < 0.004$)<br><br>1.56 ($p = 0.02$), when controlling for training in internal medicine and stated importance of small community living)	Participants were about twice as likely to leave their practice of original placement and about 50% more likely to leave rural practice than non-participants.	Observational study<br><br>Selection bias due to selective participation in the NHSC not controlled for. However, the study does controls for a variable ("importance of small community living") that is likely to partially capture health workers' preferences to work in underserved areas before financial-incentive programs could have influenced those preferences.

Program name	Study	Study outcomes	Study design	Sample criteria and sample size	Data sources	Outcome measures and study findings	Conclusions	Methodological limitations
NHSC	Pathman et al. 1994 [17]	*Program effects:* Retention (in the same underserved area)<br>Retention (in any underserved area)	Retrospective cohort study	Physicians who responded to a mail survey in 1990 and who had worked during any part of 1981 in one of 192 practices that had been included in a stratified random sample of non-metropolitan practices receiving external subsidies (N = 202)	Mail survey in 1990	*Proportion of physicians who still worked in a non-metropolitan county in 1990: NHSC vs. non-NHSC physicians:* 24% vs. 52% ($p < 0.001$)<br>*Proportion of physicians who still worked in the same practice in a non-metropolitan county in 1990: NHSC vs. non-NHSC physicians:* 13% vs. 44% ($p < 0.001$)	From 1981 through 1990, participants were about half as likely to remain in a non-metropolitan area and about three times less likely to remain in the same practice than non-participants.	Observational study<br>No control of confounding<br>Selection bias due to selective participation in the NHSC not controlled for

Program name	Study	Study outcomes	Study design	Sample criteria and sample size	Data sources	Outcome measures and study findings	Conclusions	Methodological limitations
NHSC	Pathman et al. 1994 [18]	*Program results:* Participant satisfaction<br><br>*Program effects:* Retention (in the same underserved area)<br><br>Participant satisfaction	Retrospective cohort study	All primary care NHSC physicians who started their practice obligation in a rural HPSA from 1987 to 1990 (N = 417)<br><br>Stratified random sample of non-NHSC physicians comparable in age and career stage who began working in a rural HPSA from 1987 to 1990 (N = 206)	Mail survey in 1991	*Proportion of physicians who remained at first practice site: NHSC vs. non-NHSC physicians:* 57% vs. 70% (OR = 0.56, p = 0.004) after 3 years and 1 month<br><br>21% vs. 52% (OR = 0.25, p < 0.001) after 5 years and 1 month<br><br>aOR = 0.41 (p = 0.01) after 5 years and 1 month, when controlling for "physician discipline (osteopath vs. allopath", "physician specialty ("family medicine vs. other)", "physician initial underserved-area retention plans (10 years or longer vs. less than 10 years)", "practice percentage of minority patients", "county population", county status (metropolitan vs. nonmetropolitan)", "county per capita income", and county primary care physician-to-population ratio"<br><br>*Mean satisfaction score: NHSC physicians:* Across 18 dimensions of satisfaction, 9 mean satisfaction scores were between "dissatisfied" and "neutral", 8 were between "neutral" and "satisfied", and one ("Caring for needy patients") was between "satisfied" and "very satisfied".	Five years after starting work at a practice site, participants were less than half as likely as non-participants to have remained at the site.<br><br>Across a number of dimensions, participants reported lower satisfaction with their stay in an underserved area than non-participants.	Observational study<br><br>Selection bias due to selective participation in the NHSC not controlled for. However, the study does control for a variable ("physician initial underserved-area retention plans") that is likely to partially capture health workers' preferences to work in underserved areas before financial-incentive programs could have influenced those preferences.

Program name	Study	Study outcomes	Study design	Sample criteria and sample size	Data sources	Outcome measures and study findings	Conclusions	Methodological limitations
						Mean satisfaction score: NHSC vs. non-NHSC physicians: Across 15 dimensions of satisfaction, NHSC physicians reported significantly lower satisfaction than non-NHSC physicians for "Referral Access to Consultants", "Freedom from Bureaucratic Interference", "Clinical Autonomy", "Opportunities to Achieve Professional Goals", "Earnings From Practice", "Quality Physician-Patient Relationships", "Life in a Small Community", "Climate or Geography", "Access to Cultural Activities", "Having Amenities of City Living" (all $p \leq 0.006$), and reported higher satisfaction only for "Caring for Needy Patients" ($p = 0.003$).		

Program name	Study	Study outcomes	Study design	Sample criteria and sample size	Data sources	Outcome measures and study findings	Conclusions	Methodological limitations
NHSC	Pathman and Konrad 1996 [19]	*Program results:* Retention (in the same underserved area)<br>Participant satisfaction<br>Family satisfaction	Retrospective cohort study	All primary care physicians placed through NHSC in a HPSA between 1987 and 1990 (N = 398)	Mail survey in 1991	*Proportion of physicians who remained in the practice where they had served their obligation at least one year beyond the obligation: minority vs. non-minority NHSC physicians:* 15% vs. 21% (RR = 0.71, p = 0.24)<br>*Mean satisfaction score: minority vs. non-minority NHSC physicians:* Across 17 dimensions of satisfaction, all mean satisfaction scores for both minority and non-minority physicians were between "dissatisfied" and "satisfied", with the exception of the score for "Caring for needy patients" which was between "satisfied" and "very satisfied" for both groups.<br>Minority physicians had significantly lower mean scores than their non-minority peers for "Clinical autonomy", "Opportunities to achieve professional goals", "Earnings from practice", "Opportunities for outdoor sports", "Life in a rural community", "Climate or geography", and "Ability to find compatible friends" (all $p \leq 0.04$).	Minority and non-minority participants did not differ in their retention in the practice of original placement after completion of their obligation.<br>Across a number of dimensions, minority physicians reported lower satisfaction with the stay in an underserved area (for themselves and their families) than non-minority physicians.	Observational study<br>No control of confounding

Program name	Study	Study outcomes	Study design	Sample criteria and sample size	Data sources	Outcome measures and study findings	Conclusions	Methodological limitations
						Proportion of minority vs. non-minority NHSC physicians who disagreed with the following statements about family satisfaction:<br>"[S]pouses knew what to expect when they came to their community": 38% vs. 24% (p = 0.02)<br><br>"[S]pouses were employed happily in the community": 51% vs. 32% (p = 0.004).<br><br>*Proportion of minority vs. non-minority NHSC physicians who agreed with the following statements about family satisfaction:*<br>"[S]pouses were unhappy in the community": 45% vs. 34% (p = 0.07)<br><br>"[C]hildren were happy in the community": 59% vs. 74% (p = 0.02)		

Program name	Study	Study outcomes	Study design	Sample criteria and sample size	Data sources	Outcome measures and study findings	Conclusions	Methodological limitations
NHSC	Rosenblatt et al. 1996 [20]	*Program results:*<br>Retention (in the same underserved area)<br><br>Retention (in any underserved area)<br><br>Participant satisfaction	Description of program outcomes	All physicians who graduated from medical school between 1980 and 1983, received NHSC scholarships, completed family medicine residencies, completed their practice obligation in a rural area, and responded to the survey<br>(N = 258)	Mail survey in 1994<br><br>Health Resources and Services Administration Area Resource File<br><br>Public Health Service records<br><br>American Medical Association Physician Masterfile<br><br>NHSC participant roster	*Proportion of NHSC physicians who remained in the county of original placement (an average of 6.1 years after the end of their practice obligation):*<br>25%<br><br>*Proportion of NHSC physicians who left the county of original placement to practice in a rural county:*<br>27%<br><br>*Proportions of NHSC physicians whose written accounts of their experience with the NHSC were characterized by an independent investigator as indicating a positive, neutral, ambivalent, or negative appraisal:*<br>Of 183 written comments, 41% were "either mixed or ambivalent"; 33% were "positive"; 20% were "negative"; and 6% were "neutral"	About six years after completing their obligation a quarter of participants continued to practice in the county of original placement, while about another quarter had left the original placement site to practice in another rural county.<br><br>Only 33% of participants rated their NHSC experience "positive".	Descriptive study<br><br>No control group<br><br>Duration of average retention reported but duration of individual retention not taken into account

Program name	Study	Study outcomes	Study design	Sample criteria and sample size	Data sources	Outcome measures and study findings	Conclusions	Methodological limitations
NHSC	Cullen et al. 1997 [21]	*Program results:* Retention (in the same underserved area)<br><br>Retention (in any underserved area)	Description of program outcomes	All NHSC scholarship recipients who graduated from medical school between 1975 and 1983 and were placed in a rural county (N = 6,249)	American Medical Association Physician Masterfile<br><br>NHSC participant roster	*Proportion of NHSC physicians who remained in their rural county of original NHSC placement in December 1991:*<br>13% (among those graduated from medical school in 1975-1977)<br>17% (1978-1980)<br>20% (1981-1983)<br><br>*Proportion NHSC physicians who remained in any rural county:*<br>35% (among those graduated from medical school in 1975-1977)<br>36% (1978-1980)<br>40% (1981-1983)	8-10 years after graduating from medical school, one fifth of participants remained in the county of their original placement, while two fifths remained in a rural county. 14-16 years after graduation, these proportions had fallen to 13% and 35%, respectively.	Descriptive study<br><br>No control group

Program name	Study	Study outcomes	Study design	Sample criteria and sample size	Data sources	Outcome measures and study findings	Conclusions	Methodological limitations
NHSC	Xu et al. 1997 [22]	*Program effects:* Provision of care (in any underserved area)	Retrospective cohort study	Random sample of physicians who graduated from allopathic or osteopathic medical schools in 1983 or 1984 and whose self-identified specialty was family practice, general practice, general internal medicine, or general pediatrics (N = 1,588)	Mail survey in 1994	*Odds ratio of physicians' practice in underserved areas: NHSC vs. non-NHSC physicians:* aOR = 3.7 ($p < 0.0001$), when controlling for age, sex, race/ethnicity, having grown up in an underserved area, family income as a child, strong interest in working in underserved areas prior to medical school, debt, medical school experience in an underserved area, and residency experience in an underserved area	Holding other factors constant, participants were significantly more likely to practice in an underserved area ten years after graduating from medical school than non-participants.	Observational study<br><br>Selection bias due to selective participation in the NHSC not controlled for. However, the study does control for two variables (strong interest in working in underserved areas prior to medical school and having grown up in an underserved area) that are likely to partially capture health workers' preferences to work in underserved areas before financial-incentive programs could have influenced those preferences.

Program name	Study	Study outcomes	Study design	Sample criteria and sample size	Data sources	Outcome measures and study findings	Conclusions	Methodological limitations
NHSC	Xu et al. 1997 [23]	*Program effects:* Provision of care (in any underserved area)	Retrospective cohort study	Random sample of physicians who graduated from medical school in 1983 or 1984 and whose self-reported specialty was family practice, general internal medicine, or general pediatrics (N = 1,581)	Mail survey in 1993	*Percentage of underserved patients: NHSC vs. non-NHSC physicians:* 30% vs. 19%<br>*NHSC enrollment coefficient in ordinary least squares regression with the percentage of a physician's patients who were underserved as dependent variable:*[1] 7.46 (p = 0.0001), when controlling for sex, race/ethnicity, family income as a child, and growing up in an underserved area.	Participants had about one third more underserved patients than non-participants.	Observational study<br>Selection bias due to selective participation in the NHSC not controlled for
NHSC	Singer et al. 1998 [25]	*Program effects:* Retention (in the same underserved area)	Retrospective cohort study	All physicians working in a community health center during the period January 1, 1990 through September 30, 1992 (N = 2,654)	Administrative dataset at the Bureau of Primary Health Care	*Proportion of physicians who were still working at the same community health center five years after starting their contracts: NHSC vs. non-NHSC physicians* 17% vs. 36%	After five years of work in a community health center, participants were less than half as likely as non-participants to still work at the same centre.	Observational study<br>No control of confounding<br>Selection bias due to selective participation in the NHSC not controlled for

[1] In the study, underserved patients were defined as patients who the physician considered poor or whose primary insurance was Medicaid. Medicaid is a means-tested health program funded by the US federal government and the states. It covers some categories of health care expenditures of low-income individuals, including children, parents, pregnant women, and children with disabilities [24].

Program name	Study	Study outcomes	Study design	Sample criteria and sample size	Data sources	Outcome measures and study findings	Conclusions	Methodological limitations
NHSC	Rabinowitz et al. 2000 [26]	*Program effects:* Provision of care (in any underserved area)	Retrospective cohort study	Stratified random sample of all allopathic and osteopathic physicians with a primary care specialty who graduated from a US medical school in 1983 or 1984 (N = 2,955)	American Medical Association Physician Masterfile<br>NHSC participant roster<br>Mail survey in 1993	*Odds ratio of "providing substantial care to the underserved": NHSC vs. non-NHSC physicians:*<br>aOR = 2.2 (95% CI 1.6-3.0), when controlling for sex, ethnicity, family income when growing up, childhood in inner-city/rural area, strong interest in underserved practice prior to medical school, and clinical experience with underserved patients during medical school	Holding other factors constant, participants were significantly more likely to provide substantial care to the underserved than non-participants.	Observational study<br>Selection bias due to selective participation in the NHSC not controlled for. However, the study does control for two variables (strong interest in underserved practice prior to medical school and childhood in inner-city/rural area) that are likely to partially capture health workers' preferences to work in underserved areas before financial-incentive programs could have influenced those preferences.

Program name	Study	Study outcomes	Study design	Sample criteria and sample size	Data sources	Outcome measures and study findings	Conclusions	Methodological limitations
NHSC	Rabinowitz et al. 2001 [27]	*Program effects:* Provision of care (in any underserved area)	Retrospective cohort study	All physicians who graduated from Jefferson Medical College, Philadelphia, from 1978 through 1993 (N = 3,365)	Administrative dataset of the Jefferson Medical College Alumni Association<br>Jefferson Longitudinal Study of Medical Education<br>American Medical Association Physician Masterfile	*Proportion of physicians who provided primary care in a rural area in 1999: NHSC vs. non-NHSC physicians:* 24% vs. 5% ($p < 0.001$)\<br>*Odds ratio of provision of primary care in a rural areas: NHSC vs. non-NHSC physicians:* aOR = 2.6 ($p = 0.006$), when controlling for sex, expected peak income >$131,450, freshman-year plans for family practice, rural preceptorship, rural family practice clerkship location, and participation in Physician Shortage Area Program (PSAP)[2]	Holding other factors constant, participants were significantly more likely to provide primary care in a rural area than non-participants.	Observational study<br>Selection bias due to selective participation in the NHSC not controlled for
NHSC	Mofidi et al. 2002 [28]	*Program results:* Retention (in any underserved area)	Description of program outcomes	Stratified random sample of all NHSC dentists who had completed their practice obligation between 1980 and 1997 (N = 249)	Mail survey in 1998	*Proportion of NHSC dentists in 1998 who provided care to an underserved population after completion of their practice obligation:* 47%	About half of participants continued to provide care to the underserved after their obligated service.	Descriptive study<br>No control group<br>Duration of individual retention not taken into account

[2] "The PSAP recruits and selectively admits [to Jefferson Medical College] academically qualified students who grew up or lived in a rural area or small town, and who also have a firm commitment to practice the specialty of family practice in a similar area" [27].

Program name	Study	Study outcomes	Study design	Sample criteria and sample size	Data sources	Outcome measures and study findings	Conclusions	Methodological limitations
NHSC	Brooks et al. 2003 [29]	*Program effects:* Provision of care (in any rural area)	Retrospective cohort study	All of Florida's rural primary care physicians (N = 399) and a random sample of 10% of Florida's urban and suburban primary care physicians (N = 1,236)	Mail survey in 2001	*Proportion of survey respondents who ever served in the NHSC:* 13% of rural primary care physicians, 3% of suburban primary care physicians, and 3% of urban primary care physicians had ever served in the NHSC ($p < 0.01$).	The proportion of primary care doctors who had ever served in NHSC is almost four times as high in rural areas as in either urban or suburban areas.	Observational study<br>No control of confounding<br>Selection bias due to selective participation in the NHSC not controlled for
NHSC	Porterfield et al. 2003 [30]	*Program results:* Retention (in any underserved area)	Descriptive study	Stratified random sample of all NHSC health professionals who began fulfilling their practice obligation between 1980 and 1997 (N = 1,250)	Mail survey	*Proportion of NHSC participants who worked in any underserved area in 1998:* 53%<br>*Proportion of NHSC participants who worked in any underserved area in 1998 by NHSC enrolment cohort:* 46% (1980-1984 cohort) 55% (1985-1990 cohort) 68% (1991-1997 cohort)	7 to 17 years after starting to fulfill their obligation, about half of the participants still worked in an underserved area.	Descriptive study<br>No control group<br>Selection bias due to selective participation in the NHSC not controlled for

Program name	Study	Study outcomes	Study design	Sample criteria and sample size	Data sources	Outcome measures and study findings	Conclusions	Methodological limitations
NHSC	Probst et al. 2003 [31]	*Program effects:* Provision of care (in any underserved area)	Retrospective cohort study	All allopathic and osteopathic physicians practicing in South Carolina during 1998 who were not enrolled in residency training, had graduated from medical school in 1969 or later and were not currently fulfilling a NHSC practice obligation (N = 3,608)	Physician licensure and inpatient discharge files from the Office of Research and Statistics of the South Carolina Budget and Control Board<br>NHSC participant roster	*Proportion of Medicaid patients of all discharges attended: NHSC alumni vs. other physicians*<br>28% vs. 19%<br>*Odds ratio of being highly engaged in Medicaid inpatient practice (i.e., ≥ 29.95% of their discharges were Medicaid funded) in 1998: NHSC alumni vs. non-NHSC alumni physicians:*<br>aOR = 1.93 (95% CI 1.18-3.13), when controlling for physician's sex, ethnicity, medical specialty, period of graduation from medical school, medical education in South Carolina, and graduation from a non-US medical school	Holding other factors constant, participants were significantly more likely to be engaged in Medicaid inpatient practice than non-participants.	Observational study<br>Selection bias due to selective participation in the NHSC not controlled for<br>Duration of individual inpatient practice not taken into account

Program name	Study	Study outcomes	Study design	Sample criteria and sample size	Data sources	Outcome measures and study findings	Conclusions	Methodological limitations
NHSC	Holmes 2004 [32]	*Program effects:* Provision of care (in any underserved area)<br>Retention (in the same underserved area)	Retrospective cohort study	All US physicians who graduated from medical school in 1977-1979 (N = 19,253), 1982-1984 (N = 20,757), and 1987-1989 (N = 19,500)<br>First observation of practice location in 1981, 1986, and 1991 for the 1977-1979, 1982-1984, and 1987-1989 cohorts, respectively (i.e., 2-4 years after graduation from medical school)	American Medical Association Physician Masterfile<br>NHSC participant roster<br>HPSA designation from the Bureau of Primary Health Care in the Health Resources and Services Administration	*NHSC enrollment coefficients in multiple probit regression with location in community of first practice (five years after first observation of practice location) as outcome variable:*<br>Between -0.248 and -0.272 across the three graduation cohorts (all $p < 0.01$), when controlling for age, sex, and ethnicity<br>-0.466 (not sig.), -0.866 ($p < 0.01$), and -1.748 ($p < 0.01$) in the 1977-1979, 1982-1984, and 1987-1989 cohort, respectively, when controlling for age, sex, ethnicity, and the effect of self-selection into the NHSC<br>*NHSC enrollment coefficients in multiple probit regression with practice in any HPSA as outcome variable:*<br>Between 0.528 and 0.745 across the three graduation cohorts (all $p < 0.01$), when controlling for age, sex, and ethnicity<br>0.482 (not sig.), 0.745 ($p < 0.01$), 0.161 (not sig.) in the 1977-1979, 1982-1984, and 1987-1989 cohort, respectively, when controlling for age, sex, ethnicity, and the effect of self-selection into the NHSC	Participants were less likely to remain in their first practice location than non-participants, even after the effect of self-selection into the program was controlled for.<br>Participants were more likely to serve in any underserved area than non-participants. However, this effect remained significant in only one of the three graduation cohorts, once the effect of self-selection into program participation was controlled for.	Observational study<br>Duration of individual retention not taken into account<br>Exclusion restrictions (medical school characteristics) used in selection models to control for selective participation in the NHSC may not be valid.

Program name	Study	Study outcomes	Study design	Sample criteria and sample size	Data sources	Outcome measures and study findings	Conclusions	Methodological limitations
NHSC	Pathman et al. 2005 [33]	*Program impacts:* Health	Pre-post comparison	Non-HPSA counties (N = 772)<br>HPSA counties that received various levels of NHSC staffing between 1984 and 1988:<br>0 years of staffing (N = 172)<br>1-7 years of staffing (N = 293)<br>8-11 years of staffing (N = 84)<br>12-15 years of staffing (N = 71)	Health Resources and Services Administration Area Resource File<br>NHSC participation roster	*Age-adjusted all-cause mortality rates (standardized to the 1981-1983 rate for non-HPSA counties) in 1981-1983/1996-1998:*<br>Non-HPSA: 1.000/0.947<br>HPSA, 0 years staffing: 1.022/0.982<br>HPSA, 1-7 years staffing: 1.027/0.992<br>HPSA, 8-11 years staffing: 1.092/1.055<br>HPSA, 12-15 years staffing: 1.089/1.027	There were improvements in age-adjusted mortality rates in all 5 types of counties, suggesting that changes other than the program staffing were responsible for the improvements.<br>Greater relative improvements in age-adjusted mortality were seen in non-underserved counties than in all types of underserved counties with the exception of counties staffed with program participants for 12-15 years.	Observational study<br>No control of confounding by other variables that changed over time

Program name	Study	Study outcomes	Study design	Sample criteria and sample size	Data sources	Outcome measures and study findings	Conclusions	Methodological limitations
NHSC	Holmes 2005 [34]	*Program impacts:* Health system (supply of physicians in underserved areas)	Retrospective cohort study	All US physicians who graduated from medical school in 1976-1978, 1981-1983, and 1986-1988 (N = 80,184)<br>Observation of practice location in 1981 and 1986 for the 1976-1978 cohort, in 1986 and 1991 for the 1981-1983 cohort, and in 1991 and 1996 for the 1986-1988 cohort (i.e., 3-5 years after graduation from medical school).	American Medical Association Masterfile<br>NHSC participation roster	*Predicted supply of physicians in underserved areas using parameter estimates from a sequential multinomial logit model of physicians' location decisions, which controls for the effect of self-selection into the NHSC: under the status quo vs. assuming that the NHSC were eliminated:* "[E]liminating the program would decrease the supply of physicians in medically underserved communities by roughly 10% [34]."	The NHSC contributed about one tenth to the existing US physician workforce in underserved areas.	Observational study<br>Duration of individual retention not taken into account<br>Exclusion restrictions (medical school characteristics) used in selection models to control for selective participation in the NHSC may not be valid.

Program name	Study	Study outcomes	Study design	Sample criteria and sample size	Data sources	Outcome measures and study findings	Conclusions	Methodological limitations
NHSC	Pathman et al. 2006 [35]	*Program impacts:* Health system	Retrospective cohort study	All rural HPSA staffed by NHSC physicians, nurses, and/or physician assistants in 1984 and at least 3 of the preceding 5 years (N = 141)<br>All rural HPSA that had no NHSC clinician assigned from the above disciplines between 1979 and 2001 (N = 142)	American Medical Association Physician Masterfile<br>NHSC participation roster<br>Health Resources and Services Administration Area Resource File	*NHSC staffing coefficients in multiple linear regression with ratio change in non-NHSC primary care physician density from 1981 to 2001 as dependent variable:*<br>1.06 ($p < 0.01$), when controlling for population size, ethnic composition, per-capita income, poverty prevalence, youth unemployment rate, education, presence of a hospital, presence of a community or migrant health center, non-NHSC primary care physician population density at baseline, and presence of at least one non-NHSC primary care physician at baseline	Presence of a program participant increased the supply of non-participating physicians in underserved areas on average by 6 percent.	Observational study
NHSC	Rittenhouse et al. 2008 [36]	*Program effects:* Provision of care (in any underserved area)	Retrospective cohort study	All physicians active in direct patient care (in 2001-2003) who graduated from an allopathic US medical schools and completed residency training in 1970 or later (N = 412,012)	American Medical Association Physician Masterfile<br>NHSC participation roster	*Odds ratio of work in a community health center (in 2001-2003): participants in the NHSC loan repayment program vs. non-participants:*<br>aOR = 6.99 ($p < 0.001$) , when controlling for sex, year of residency completion, private vs. public medical school, and attendance of a medical school receiving Title VII funding	Holding other factors constant, participants were significantly more likely to work in a community health center than non-participants.	Observational study<br>Selection bias due to selective participation in the NHSC not controlled for

Program name	Study	Study outcomes	Study design	Sample criteria and sample size	Data sources	Outcome measures and study findings	Conclusions	Methodological limitations
Scholarship for Indian students in health sciences	Weiss et al. 1980 [37]	*Program results:* Recruitment	Description of program outcomes	All students who were supported by the scholarship between 1973 and 1977 and graduated in 1980 (N = 124)	Navajo Health Agency Office of Student Affairs records	*Proportion of participants who practiced in the Navajo Indian reservation or immediately adjacent communities after graduation:* Of 124 participants, 34 (27%) continued their education, 9 (7%) were lost to follow-up or died, 5 (4%) were unemployed and 76 (62%) were employed<br><br>Of the 76 participants available for practice, 56 (74%) worked in the Navajo Indian reservation or immediately adjacent communities.	In a program without obligation, but encouragement, to serve in specific underserved areas, three quarters of participants decided to practice in the underserved areas.	Descriptive study<br><br>No control group
Oklahoma Rural Medical Education Scholarship Loan	Holmes and Miller 1985 [38]	*Program results:* Recruitment	Description of program outcomes	All scholarship recipients from 1976 to 1985 (N = 138)	Oklahoma Physician Manpower Training Commission records	*Proportion of participants who fulfilled their practice obligation:* Of 138 students, 94 (68%) fulfilled the obligation, while 44 (32%) repaid the financial incentive.	About two thirds of participants fulfilled their obligation to practice in an underserved area.	Descriptive study<br><br>No control group

Program name	Study	Study outcomes	Study design	Sample criteria and sample size	Data sources	Outcome measures and study findings	Conclusions	Methodological limitations
Oklahoma Rural Medical Education Scholarship Loan	Lapolla et al. 2004 [39]	*Program results:* Recruitment<br><br>Retention (in the same underserved area)	Description of program outcomes	All physicians who fulfilled their practice obligation (N = 313)	Oklahoma Physician Manpower Training Commission records	*Proportion of participants who started serving or completed their practice obligation in 2000 or before:* Of 544 participants available for practice, 407 (75%) started serving or completed the obligation and 138 (25%) repaid the financial incentive.[3]<br><br>*Proportion of participants who remained in the original placement community upon completion of their practice obligation:* Of 313 students, 167 (53%) remained in the original placement community, 91 (29%) relocated to another community in Oklahoma, and 55 (18%) relocated to another state.	One quarter of the participants fulfilled the obligation to practice in an underserved area.<br><br>About half of the participants remained in the placement community upon completion of the obligation.	Descriptive study<br><br>No control group<br><br>Duration of individual retention not taken into account

[3] The true absolute numbers may be slightly different, because the numbers in this table were derived from percentages that are shown rounded to the first integer in the source study [39].

Program name	Study	Study outcomes	Study design	Sample criteria and sample size	Data sources	Outcome measures and study findings	Conclusions	Methodological limitations
NHSC<br><br>Indian Health Service Corps<br><br>US state service-requiring scholarships<br><br>State loan repayment programs<br><br>Practice and hospital-sponsored direct financial incentives	Pathman et al. 2000 [40]	*Program effects:* Provision of care (in any underserved area)	Retrospective cohort study	Stratified random sample of all physicians who graduated from US medical schools in either 1988 or 1992 and were listed four years after graduation with a principal specialty of family practice, general internal medicine or general pediatrics (N = 468)	American Medical Association Physician Masterfile	*Proportions of financial-incentive program participants vs. non-participants who practiced in any rural area in 1999:*<br>33% vs. 7% ($p < 0.001$)<br><br>*Average proportion of Medicaid and uninsured patients of all patients who are cared for by participants vs. non-participants in 1999:*<br>54% vs. 29% ($p < 0.001$)<br><br>The positive association of participation with practice in rural areas and with the proportion of Medicaid and uninsured patients remained significant "while controlling for selected characteristics of physicians".	In comparison to non-participants, participants in financial-incentive programs were about five times more likely to practice in rural areas and 85% more likely to care for underserved populations.	Observational study<br><br>Selection bias due to selective participation in financial-incentive programs not controlled for

Program name	Study	Study outcomes	Study design	Sample criteria and sample size	Data sources	Outcome measures and study findings	Conclusions	Methodological limitations
New South Wales Department of Health Rural Resident Medical Officer Program (Cadetship Program)	Dunbabin et al. 2006 [41]	*Program results:* Recruitment<br><br>Retention (in any the same underserved area)	Description of program outcomes	All medical students who accepted the scholarship between 1989 and 2004 and should have graduated from medical school in 2004 or before (N = 157)<br><br>All medical students who accepted the scholarship between 1989 and 1998, graduated from medical school, and completed their rural service (N = 82)	New South Wales Rural Doctors Network records<br><br>Medical Directory of Australia<br><br>Mail survey in 2004	*Proportion of participants (1989-2004 cohort) who started serving or completed their practice obligation in 2004 or before:* Of 157 participants, 4 (3%) did not graduate from medical school.<br><br>Of the 153 participants who graduated from medical school, 133 (87%) started serving or completed the obligation and 20 (13%) withdrew from the program.<br><br>*Proportion of participants (1989-1998 cohort) who completed their rural service and (in 2004) practiced in a rural community:* Of 82 former participants, 35 (43%) worked in a rural area (compared to 21% of all medical practitioners nationally).	About nine tenths of participants fulfilled their obligation to practice in a rural area.<br><br>Retention in rural communities after completion of the obligation was substantial.	Descriptive study<br><br>No control group<br><br>Duration of individual retention not taken into account

Program name	Study	Study outcomes	Study design	Sample criteria and sample size	Data sources	Outcome measures and study findings	Conclusions	Methodological limitations
Community Scholarship Program (CSP)<br><br>Health Sciences Scholarship Program (HSSP)<br><br>Recruitment and Retention Community Program (RRCP)<br><br>State Loan Repayment Program (SLRP)	Jackson et al. 2003 [42]	*Program results:* Recruitment<br><br>Participant satisfaction<br><br>*Program effects:* Retention (in the same underserved area)<br><br>Participant satisfaction	Retrospective cohort study	All participants in at least 1 of the 4 incentive programs who completed at least 1 year of their practice obligation (N = 105 for study of program result, N = 44 for study of program effect)<br><br>All primary care physicians who graduated from US medical schools and were practicing in West Virginia counties defined as "rural" by both the federal Office of Management and Budget and the West Virginia Rural Health Education Partnership (N = 107)	West Virginia Board of Medicine licensure files<br><br>West Virginia School of Osteopathic Medicine<br><br>Mail survey in 2002	*Proportion of participants who started or completed their practice obligation in 2002 or before:* Of 105 participants available for practice, 82 (78%) started or completed the obligation and 23 (22%) repaid the financial incentive.<br><br>*Comparison of the proportion of participants vs. the proportion of all other primary care physicians who were still practicing at their first practice site in 2002:* "Obligated physicians were less likely to leave their service sites during the first 4 years of practice than were nonobligated physicians. After obligations were completed and physicians were free to leave, retention dropped into the range seen among nonobligated physicians."<br><br>After 4 years, 32% of all participants were no longer at their first practice site, compared with 38% of all other primary care physicians (RR = 0.84, p = 0.475).[4]	More than three quarters of participants fulfilled their obligation to practice in an underserved area.<br><br>Retention in the first practice site was not significantly different between program participants and non-participants.<br><br>The majority of participants in one of the four evaluated financial-incentive programs were satisfied with their experience.	Observational study<br><br>No control of confounding<br><br>Selection bias due to selective participation in financial-incentive programs not controlled for

[4] Calculated using information available in the article [42].

Program name	Study	Study outcomes	Study design	Sample criteria and sample size	Data sources	Outcome measures and study findings	Conclusions	Methodological limitations
						Proportion of financial-incentive program participants vs. non-participants who "agreed that clinical work was personally rewarding":<br>98% vs. 85% (p = 0.02)<br><br>*Proportions of participants who were dissatisfied with program personnel:*<br>Across the different evaluated programs "one third to half of recipients of all programs felt they had too little contact, assistance, and responsiveness" from program personnel.<br><br>*Proportion of participants who would participate again in the same program:*<br>Of 41 program participants who provided a valid answer when asked whether "they would sign up for their financial incentive program again", 30 (73%) answered "definitely yes" or "probably yes", while the remainder answered "definitely not" or "probably not".		

Program name	Study	Study outcomes	Study design	Sample criteria and sample size	Data sources	Outcome measures and study findings	Conclusions	Methodological limitations
20 US state service-requiring scholarship programs<br>12 state loan service-option programs<br>24 state loan repayment programs<br>6 state direct financial-incentive programs for residents<br>7 state direct financial-incentive programs for fully trained health professionals	Pathman et al. 2004 [43]	*Program results:* Participant satisfaction<br>Family satisfaction<br>*Program effects:* Retention (in the same underserved area)	Description of program outcomes<br>Retrospective cohort study	All primary care physicians serving or having served their practice obligation in 1991 or 1996 (N = 330)<br>Stratified random sample of all graduates of US allopathic and osteopathic medical schools in 1988 and 1992 who 4 years after graduation were in primary care practice in the US and were not obligated to serve in a specific location (N = 468)	American Medical Association Physician Masterfile<br>Records of the individual state programs<br>1999 US census<br>Health Resources and Services Administration Area Resource File<br>Mail survey in 1998 and 1999	*Proportion of program participants who completed their practice obligation in 2004 or before:*<br>45% (average of service-option loan programs)<br>67% (average of scholarship programs)<br>93% (average of all other programs included in the study)<br>*Proportion of program participants who repaid the financial incentive by 2004:*<br>49% (average of service-option loan programs)<br>27% (average of scholarship programs)<br>2% (average of all other programs included in the study)<br>*Hazard ratio of retention at first practice site: program participants vs. program non-participants:*<br>0.70 (p = 0.029)<br>0.75 (p = 0.080), when controlling for age, sex, medical specialty, and marital status	Programs that enrolled physicians after graduation from medical school achieved higher obligation completion ratios than programs that enrolled students during medical school.<br>Participants were about 25% less likely to remain at their site of first practice than non-participants. However, this difference was not statistically significant, when age, sex, medical specialty and marital status were controlled for.	*Program outcome:* Descriptive study<br>*Program effect:* Observational study<br>Selection bias due to selective participation in financial-incentive programs not controlled for

Program name	Study	Study outcomes	Study design	Sample criteria and sample size	Data sources	Outcome measures and study findings	Conclusions	Methodological limitations
						Proportion of participants who were satisfied with their program placement:<br>More than 70% of obligated physicians felt "a sense of belonging to the community"; more than 80% were "satisfied with practice"; and more than 90% found their "work rewarding". All three proportions were lower among nonobligated physicians (all $p \leq 0.05$).<br><br>*Proportion of family members who were satisfied with program placement:*<br>The spouses of obligated physicians did not differ significantly from the spouses of nonobligated physicians in their satisfaction with the community (more than 50%) or with their work (more than 70%). The children of obligated physicians did not differ significantly from children of nonobligated physicians in their satisfaction with the community (more than 80%).<br><br>*Proportion of participants who would participate again in the same program:*<br>90% of obligated physicians would "likely" or "definitely" participate again in their financial-incentive program.	The majority of participants in one of the evaluated financial-incentive programs were satisfied with their experience; their spouses were significantly less satisfied.	

Program name	Study	Study outcomes	Study design	Sample criteria and sample size	Data sources	Outcome measures and study findings	Conclusions	Methodological limitations
Friends of Mosvold Scholarship Scheme (FOMSS)	Ross 2007 [44]	*Program results:* Recruitment	Description of program outcomes	All individuals who participated in the program between 1999 and 2002 and graduated from a health care education program before 2006 (N = 24)	FOMSS records	*Proportion of participants who started serving or completed their practice obligation in 2006 or before:* Of 24 participants who graduated from a health care education program, 1 (4%) died and 3 (13%) pursued further education or training. Of 20 participants available for service, 20 (100%) started serving or completed the obligation.	All available participants fulfilled their obligation to practice in an underserved area.	Descriptive study

OR = odds ratio, aOR = adjusted odds ratio, RR = risk ratio. The term underserved area in the table encompasses a specific underserved area, any underserved area, and underserved populations. The sample size N refers to the largest number of people included in an analysis of program outcomes reported in a study. Some outcome analyses in the same study may use samples that are smaller than N, for instance, because data on a particular outcome were not available for all individuals. WWII = Second World War, HPSA = Health Professional Shortage Area, not sig. = not significant at the 5% level.

References

1. Fitz RH, Mawardi BH, Wilber J: **Scholarships for rural medicine. The Commonwealth Fund experience with a pre-World War II indenture program**. *Trans Am Clin Climatol Assoc* 1977, **88**:191-196.
2. Mason HR: **Effectiveness of student aid programs tied to a service commitment**. *J Med Educ* 1971, **46**(7):575-583.
3. Bradbury SF: **The North Carolina Medical Care Commission: evaluation of the Rural Loan Program by recipients of medical and dental loans**. *N C Med J* 1963, **24**:489-491.
4. Navin TR, Nichols AW: **Evaluation of the Arizona Medical Student Exchange Program**. *J Med Educ* 1977, **52**(10):817-823.
5. Bass M, Copeman WJ: **An Ontario solution to medically underserviced areas: evaluation of an ongoing program**. *Canadian Medical Journal* 1975, **113**:403-407.
6. Anderson M, Rosenberg MW: **Ontario's underserviced area program revisited: an indirect analysis**. *Soc Sci Med* 1990, **30**(1):35-44.
7. Inoue K, Hirayama Y, Igarashi M: **A medical school for rural areas**. *Med Educ* 1997, **31**(6):430-434.
8. Inoue K, Matsumoto M, Sawada T: **Evaluation of a medical school for rural doctors**. *J Rural Health* 2007, **23**(2):183-187.
9. Matsumoto M, Inoue K, Kajii E: **A contract-based training system for rural physicians: follow-up of Jichi Medical University graduates (1978-2006)**. *J Rural Health* 2008, **24**(4):360-368.
10. Matsumoto M, Inoue K, Kajii E: **Characteristics of medical students with rural origin: implications for selective admission policies**. *Health Policy* 2008, **87**(2):194-202.
11. Matsumoto M, Inoue K, Kajii E: **Long-term effect of the home prefecture recruiting scheme of Jichi Medical University, Japan**. *Rural Remote Health* 2008, **8**(3):930.
12. Woolf MA, Uchill VL, Jacoby I: **Demographic factors associated with physician staffing in rural areas: the experience of the National Health Service Corps**. *Med Care* 1981, **19**(4):444-451.
13. Stamps PL, Kuriger FH: **Location decisions of National Health Service Corps physicians**. *Am J Public Health* 1983, **73**(8):906-908.
14. Stone VE, Brown J, Sidel VW: **Decreasing the field strength of the National Health Service Corps: will access to care suffer?** *J Health Care Poor Underserved* 1991, **2**(3):347-358.
15. Brown J, Stone V, Sidel VW: **Decline in NHSC physicians threatens patient care**. *Am J Public Health* 1990, **80**(11):1395-1396.
16. Pathman DE, Konrad TR, Ricketts TC, 3rd: **The comparative retention of National Health Service Corps and other rural physicians. Results of a 9-year follow-up study**. *JAMA* 1992, **268**(12):1552-1558.
17. Pathman DE, Konrad TR, Ricketts TC, 3rd: **Medical education and the retention of rural physicians**. *Health Serv Res* 1994, **29**(1):39-58.
18. Pathman DE, Konrad TR, Ricketts TC, 3rd: **The National Health Service Corps experience for rural physicians in the late 1980s**. *JAMA* 1994, **272**(17):1341-1348.

19. Pathman DE, Konrad TR: **Minority physicians serving in rural National Health Service Corps sites**. *Med Care* 1996, **34**(5):439-454.
20. Rosenblatt RA, Saunders G, Shreffler J, Pirani MJ, Larson EH, Hart LG: **Beyond retention: National Health Service Corps participation and subsequent practice locations of a cohort of rural family physicians**. *J Am Board Fam Pract* 1996, **9**(1):23-30.
21. Cullen TJ, Hart LG, Whitcomb ME, Rosenblatt RA: **The National Health Service Corps: rural physician service and retention**. *J Am Board Fam Pract* 1997, **10**(4):272-279.
22. Xu G, Veloski JJ, Hojat M, Politzer RM, Rabinowitz HK, Rattner S: **Factors influencing physicians' choices to practice in inner-city or rural areas**. *Acad Med* 1997, **72**(12):1026.
23. Xu G, Fields SK, Laine C, Veloski JJ, Barzansky B, Martini CJ: **The relationship between the race/ethnicity of generalist physicians and their care for underserved populations**. *Am J Public Health* 1997, **87**(5):817-822.
24. **Medicaid** [http://www.cms.hhs.gov/home/medicaid.asp]
25. Singer JD, Davidson SM, Graham S, Davidson HS: **Physician retention in community and migrant health centers: who stays and for how long?** *Med Care* 1998, **36**(8):1198-1213.
26. Rabinowitz HK, Diamond JJ, Veloski JJ, Gayle JA: **The impact of multiple predictors on generalist physicians' care of underserved populations**. *Am J Public Health* 2000, **90**(8):1225-1228.
27. Rabinowitz HK, Diamond JJ, Markham FW, Paynter NP: **Critical factors for designing programs to increase the supply and retention of rural primary care physicians**. *JAMA* 2001, **286**(9):1041-1048.
28. Mofidi M, Konrad TR, Porterfield DS, Niska R, Wells B: **Provision of care to the underserved populations by National Health Service Corps alumni dentists**. *J Public Health Dent* 2002, **62**(2):102-108.
29. Brooks RG, Mardon R, Clawson A: **The rural physician workforce in Florida: a survey of US- and foreign-born primary care physicians**. *J Rural Health* 2003, **19**(4):484-491.
30. Porterfield DS, Konrad TR, Porter CQ, Leysieffer K, Martinez RM, Niska R, Wells B, Potter F: **Caring for the underserved: current practice of alumni of the National Health Service Corps**. *J Health Care Poor Underserved* 2003, **14**(2):256-271.
31. Probst JC, Samuels ME, Shaw TV, Hart GL, Daly C: **The National Health Service Corps and Medicaid inpatient care: experience in a southern state**. *South Med J* 2003, **96**(8):775-783.
32. Holmes GM: **Does the National Health Service Corps improve physician supply in underserved locations?** *Eastern Economic Journal* 2004, **30**(4):563-581.
33. Pathman DE, Fryer GE, Green LA, Phillips RL: **Changes in age-adjusted mortality rates and disparities for rural physician shortage areas staffed by the National Health Service Corps: 1984-1998**. *J Rural Health* 2005, **21**(3):214-220.
34. Holmes GM: **Increasing physician supply in medically underserved areas**. *Labour Economics* 2005, **12**:697-725.

35. Pathman DE, Fryer GE, Jr., Phillips RL, Smucny J, Miyoshi T, Green LA: **National Health Service Corps staffing and the growth of the local rural non-NHSC primary care physician workforce**. *J Rural Health* 2006, **22**(4):285-293.
36. Rittenhouse DR, Fryer GE, Jr., Phillips RL, Jr., Miyoshi T, Nielsen C, Goodman DC, Grumbach K: **Impact of Title VII training programs on community health center staffing and National Health Service Corps participation**. *Ann Fam Med* 2008, **6**(5):397-405.
37. Weiss LD, Wiese WH, Goodman AB: **Scholarship support for Indian students in the health sciences: an alternative method to address shortages in the underserved area**. *Public Health Rep* 1980, **95**(3):243-246.
38. Holmes JE, Miller DA: **A study of 138 return service scholarship applications awarded by the Oklahoma Physician Manpower Training Commission**. *J Okla State Med Assoc* 1985, **78**(10):384-388.
39. Lapolla M, Brandt EN, Jr., Barker A, Ryan L: **State public policy: the impacts of Oklahoma's physician incentive programs**. *J Okla State Med Assoc* 2004, **97**(5):190-194.
40. Pathman DE, Konrad TR, King TS, Spaulding C, Taylor DH: **Medical training debt and service commitments: the rural consequences**. *J Rural Health* 2000, **16**(3):264-272.
41. Dunbabin JS, McEwin K, Cameron I: **Postgraduate medical placements in rural areas: their impact on the rural medical workforce**. *Rural Remote Health* 2006, **6**(2):481.
42. Jackson J, Shannon CK, Pathman DE, Mason E, Nemitz JW: **A comparative assessment of West Virginia's financial incentive programs for rural physicians**. *J Rural Health* 2003, **19 Suppl**:329-339.
43. Pathman DE, Konrad TR, King TS, Taylor DH, Jr., Koch GG: **Outcomes of states' scholarship, loan repayment, and related programs for physicians**. *Med Care* 2004, **42**(6):560-568.
44. Ross AJ: **Success of a scholarship scheme for rural students**. *S Afr Med J* 2007, **97**(11):1087-1090.
